# A dual promoter system regulating λ DNA replication initiation

**DOI:** 10.1093/nar/gku103

**Published:** 2014-02-05

**Authors:** Paweł Olszewski, Anna Szambowska, Sylwia Barańska, Magdalena Narajczyk, Grzegorz Węgrzyn, Monika Glinkowska

**Affiliations:** ^1^Department of Molecular Biology, University of Gdańsk, Wita Stwosza 59, 80-308 Gdańsk, Poland, ^2^Laboratory of Molecular Biology (affiliated with the University of Gdańsk), Institute of Biochemistry and Biophysics, Polish Academy of Sciences, Wita Stwosza 59, 80-308 Gdańsk, Poland and ^3^Laboratory of Electron Microscopy, Faculty of Biology, University of Gdańsk, Wita Stwosza 59, 80-308 Gdańsk, Poland

## Abstract

Transcription and DNA replication are tightly regulated to ensure coordination of gene expression with growth conditions and faithful transmission of genetic material to progeny. A large body of evidence has accumulated, indicating that encounters between protein machineries carrying out DNA and RNA synthesis occur *in vivo* and may have important regulatory consequences. This feature may be exacerbated in the case of compact genomes, like the one of bacteriophage λ, used in our study. Transcription that starts at the rightward *p*_R_ promoter and proceeds through the λ *origin* of replication and downstream of it was proven to stimulate the initiation of λ DNA replication. Here, we demonstrate that the activity of a convergently oriented *p*_O_ promoter decreases the efficiency of transcription starting from *p*_R_. Our results show, however, that a lack of the functional *p*_O_ promoter negatively influences λ phage and λ-derived plasmid replication. We present data, suggesting that this effect is evoked by the enhanced level of the *p*_R_-driven transcription, occurring in the presence of the defective *p*_O_, which may result in the impeded formation of the replication initiation complex. Our data suggest that the cross talk between the two promoters regulates λ DNA replication and coordinates transcription and replication processes.

## INTRODUCTION

Although biochemical reactions leading to DNA synthesis during the replication process, one of crucial biological phenomena occurring in every organism, have been described in details, the regulation of initiation of this process in prokaryotic and eukaryotic cells is still insufficiently understood. Interestingly, considerable similarities seem to exist in some regulatory reactions between prokaryotic and eukaryotic systems (1–3). One of the biggest contrasts between the initiation of DNA replication in prokaryotic and eukaryotic cells relies on the different nature of the replication start sites in these systems, which constitute discrete sequences in the first case, and poorly defined DNA regions in the latter. Despite this discrepancy, it has been suggested for many prokaryotic and eukaryotic systems (for example: bacteriophage λ, *Escherichia coli* and metazoans) that transcriptional activity of the neighboring region may have a large impact, both adverse and advantageous, on the function of *origins* of replication (4). It was also suggested that a cross talk between DNA replication and gene expression is one of the principles driving evolutionary optimization of genome organization, which enables correlation of transcription and replication during environmental and developmental changes (5,6). Thus, understanding the interplay between transcription and replication regulatory elements is a task of general biological importance.

**Figure 1.**
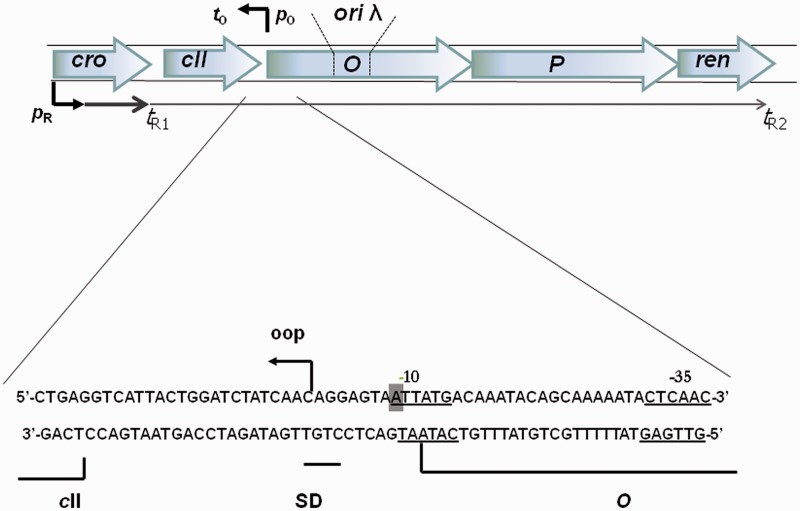
A map of bacteriophage λ replication region, which corresponds to the sequence present in a typical λ plasmid. Genes are shown in frames. The *p*_R_ and *p*_O_ promoters are marked by arrows, with arrowheads indicating direction of transcription. Positions of *t*_R1_, *t*_R2_ and *t*_O_ terminators, and of *ori*λ (in the middle of the *O* gene) are indicated. Below the map, the sequence of the *p*_O_ promoter region is shown and the transcription start site (arrow) and −10 and −35 sequences (underlined) of *p*_O_ are marked. Position of the *p*_O_− mutation (A→T transversion) is highlighted by a gray box. This mutation is separated by 2 bp from the first codon of the *O* gene.

Bacteriophage λ has served for decades as a model virus in molecular biology studies, especially in research on crucial biological processes like gene expression regulation and DNA replication (7,8). A starting point for bacteriophage λ DNA replication is marked by binding of the λO initiatior protein to four 19 bp repeats (iterons) (Figure 1) (9,10). λO is dimeric in solution, but on binding to the λ origin, dimers bound to neighboring iterons interact to form a higher-order structure, called O-some, around which DNA is wrapped (10). Assembly of this structure governs the subsequent series of reactions resulting in formation of the functional replication complex. The λP-DnaB protein complex joins initially to the O-some, forming the λO-λP-DnaB preprimosomal complex (10,11), in which activity of DnaB helicase is inhibited by the presence of the λP protein (12,13). Release of the helicase activity, necessary for propagation of replication forks, requires a coordinated action of heat-shock proteins: DnaK, DnaJ and GrpE (11,14,15) and remodeling of the preprimosome (16).

A crucial role in the regulation of the frequency of DNA replication initation at *ori*λ is played by transcription starting at the *p*_R_ promoter (17,18). This transcription event provides mRNA for main replication proteins, λO and λP, but even if these proteins are supplied *in trans*, activity of RNA polymerase in the λ *origin* region is still necessary and ensures efficient initiation of DNA replication at *ori*λ (19,20). This phenomenon, called transcriptional activation of *ori*λ, most probably operates via changes in DNA topology and is connected with preprimosome assembly and helicase loading (21). Activity of *p*_R_ was also demonstrated to affect directionality of the λ DNA replication using both *in vitro* and *in vivo* approaches (22,23). Recently, a direct interaction between the λO protein and RNA polymerase was shown *in vitro* (24). This interaction enhances formation of a stable nucleoprotein complex between the replication initiator and its recognition sites within the origin region (24). Transcriptional activation of *ori*λ is therefore considered as the main regulatory process influencing both efficiency and directionality of λ DNA replication (25). On the other hand, transcription starting from the *p*_R_ promoter was shown to stimulate degradation of the λO protein by the ClpX/ClpP protease complex, thus decreasing stability of the O-some structure, unless λO was embedded in the preprimosomal complex together with λP and DnaB (26).

Another promoter present in the λ replication region, called *p*_O_ (Supplementary Figure S1), which serves as a starting-point for synthesis of a short leftward transcript (*oop*), was previously suggested to influence the replication initiated at *ori*λ (27). It was speculated that *oop* may serve as a primer for the replication forks proceeding leftward. However, subsequent experiments showed that the DnaG primase function is sufficient for production of primers during λ plasmid replication (28,29), and that *oop* RNA is an antisense RNA for a transcript produced from the *cII* gene, involved in the establishment of lysogeny (30,31). Therefore, the hypothesis about involvement of *oop* RNA in λ DNA replication was considered unlikely. On the other hand, results of our subsequent studies indicated that the activity of *p*_O_ plays an important role in the regulation of replication initiated at *ori*λ. Mutation in the −10 region of *p*_O_ (Figure 1), resulting in inactivation of this promoter, caused a significant decrease in the λ plasmid copy number and the rate of the λ plasmid DNA synthesis (32). Moreover, two DNA sequences resembling DnaA box consensus were identified downstream of the *p*_O_ promoter, and protection of these sites by DnaA was confirmed in *in vitro* footprinting experiments (33). However, the exact role of *p*_O_ in the regulation of λ DNA replication remained obscure. Interestingly, recent discoveries of direct interactions between RNA polymerase and both λO (24) and DnaA (34), strongly suggest a cross talk between transcription and replication machineries at replication *origin* regions. Thus, these findings underscore the importance of promoters located in the vicinity of *ori*λ.

In this work, we studied the role of the *p*_O_ promoter activity in λ DNA replication and the interdependence between *p*_R_ and *p*_O_, and demonstrated its impact on the λ plasmid replication *in vivo*. Results presented in this work imply that interplay between transcription elements may strongly influence formation of replication complexes, which implicates their role as a precise device coordinating DNA replication with metabolic status of the cell.

## MATERIALS AND METHODS

### Bacterial strains, plasmids and bacteriophages and oligonucleotides

Bacterial strains used in this study are described in the Supplementary Material (Supplementary Table S1). Plasmids and bacteriophages are listed in Supplementary Table S2 and oligonucleotides in Supplementary Table S3. All genetic manipulations were described in the Supplementary Material.

### Plasmid maintenance

Plasmid maintenance was investigated according to the previously described method (35).

### Efficiency of transformation by the two-*origin* plasmids of bacteria bearing a helper plasmid

*Escherichia coli* C600 or C600polA1 strains, bearing a hybrid ColE1-λ helper plasmid, pLamberA, were transformed by a series of plasmids bearing various insertions between *p*_O_ and *ori*λ. Efficiency of transformation was estimated by determining a number of transformants obtained per 1 μg of DNA used in the experiment.

### Determination of plasmid copy number

Plasmid copy number in *E. coli* cells was measured as described earlier (36).

### Measurement of β-galactosidase activity

Activity of β-galactosidase was measured according to Miller (37). Detailed description is provided in the Supplementary Material.

### Protein purification

λO and λP proteins were purified from *E. coli* strain MM294 bearing pEW1 and pGP1-2 plasmids (Supplementary Table S2). The purification procedures have been described previously (38).

### Preparation of Fraction II and *in vitro* DNA replication

Fraction II and the *in vitro* replication assay were prepared essentially according to a procedure described by Fuller *et al.* (39). Detailed description was included in the Supplementary Material.

### Analysis of directionality of plasmid DNA replication

Directionality of λ plasmid DNA replication was studied by analysis of replication intermediates separated during two-dimensional agarose gel electrophoresis (2D-AGE) according to Viguera *et al.* (40), with modifications described by Srutkowska *et al.* (41). *In silico* prediction of possible results of 2D-AGE was performed using the method described previously (42).

### Electrophoretic mobility shift assay

Fifty nanograms of a Cy5-labeled DNA fragment (52 bp long) encompassing the *p*_O_ promoter sequence was mixed with rising concentrations of RNA polymerase in a buffer containing 25 mM Hepes-KOH, pH 7.6, 100 mM potassium glutamate, 5 mM magnesium acetate, 4 mM dithiothreitol (DTT), 2% Triton X-100 and 50 ng/μl poly dI-dC, in 20 μl of total volume. The samples were incubated for 10 min at 37°C, and subsequently resolved electrophoretically in 5% polyacrylamide gel (19:1 acrylamide:bisacrylamide, 0.5 × TBE, 2.5% glycerol) running in 0.5 × TBE (45 mM Tris-borate/1 mM EDTA) at 9 V/cm at 4°C. DNA was visualized using GE Healthcare Typhoon 9200 scanner.

### Density shift experiments

Density shift experiments were carried on as described earlier (43). Full description is included in the Supplementary Material.

## RESULTS

### Effect of the *p*_O_− mutation on replication of bacteriophage λ DNA

Our previous studies demonstrated that the single base substitution in the −10 region of the *p*_O_ promoter (Figure 1) decreases the efficiency of replication of plasmids derived from bacteriophage λ (32). This mutation is located close to, but not within, the start codon of the *O* gene, coding for the λO replication initiator protein (Figure 1). However, when the effect of the *p*_O_− mutation on the *O* expression was tested, a slight increase rather than a decrease in the level of the replication initiator protein could be detected [(44), and our unpublished observations]. Therefore, an impairment in the replication of the λ plasmid bearing this defective *p*_O_ promoter cannot be explained by changes in intracellular amount of the λO protein. Moreover, a significantly (at least several times) increased levels of the *p*_O_-derived *oop* RNA (arising from dysfunction of the *pcnB* gene, and resultant impaired RNA polyadenylation causing an increased stability of this short transcript) did not influence considerably copy number of both wild-type λ plasmid and its *p*_O_− derivative (44). Thus, any significant effects of *in trans* action of the *p*_O_-initiated transcript on the regulation of replication from *ori*λ are also unlikely. In this light, a role for the *p*_O_ promoter activity *per se* appeared the most probable hypothesis.

To further investigate the physiological significance of the *p*_O_-mediated effects on *ori*λ function, the influence of the *p*_O_− mutation on λ phage DNA replication was assessed using density shift experiment, according to the previously described method (43,45). This method allows for distinction between newly synthesized DNA molecules, which incorporate ‘heavy’ isotopically labeled nucleotides, and parental (‘light’) DNA strands. Bacteriophage λ DNA replicates according to two modes (8). Early after infection, θ (circle to circle) replication occurs and after a few rounds it is switched to the σ (rolling circle) mode, producing long concatameric λ genomes. Results of earlier studies demonstrated that transcription from the *p*_R_ promoter influences directionality of λ DNA replication, and this, in turn, affects the timing of the switch from θ to σ mode. Namely, unidirectional replication, resulting from insufficient transcriptional activation of the *origin*, was shown to cause earlier occurrence of the σ mode (46,47). Density shift experiments allowed to assess the influence of the *p*_O_ promoter activity on both the efficiency of replication and the timing of the switch from θ to σ mode. To achieve this, *E. coli* cells, growing in a ‘light’ minimal medium were infected with λ phage and immediately transferred to a ‘heavy’ minimal medium, containing [^13^C]glucose and [^15^N]H_4_Cl. DNA isolation, followed by ultracentrifugation in CsCl density gradient revealed the presence of products of subsequent phage DNA replication rounds: heavy-light DNA molecules, containing one heavy and one light strand, and full-heavy molecules. Appearance of full-heavy DNA early after infection would indicate either a rapid switch from θ to σ mode of replication or enormously frequent initiation of θ replication.

**Figure 2.**
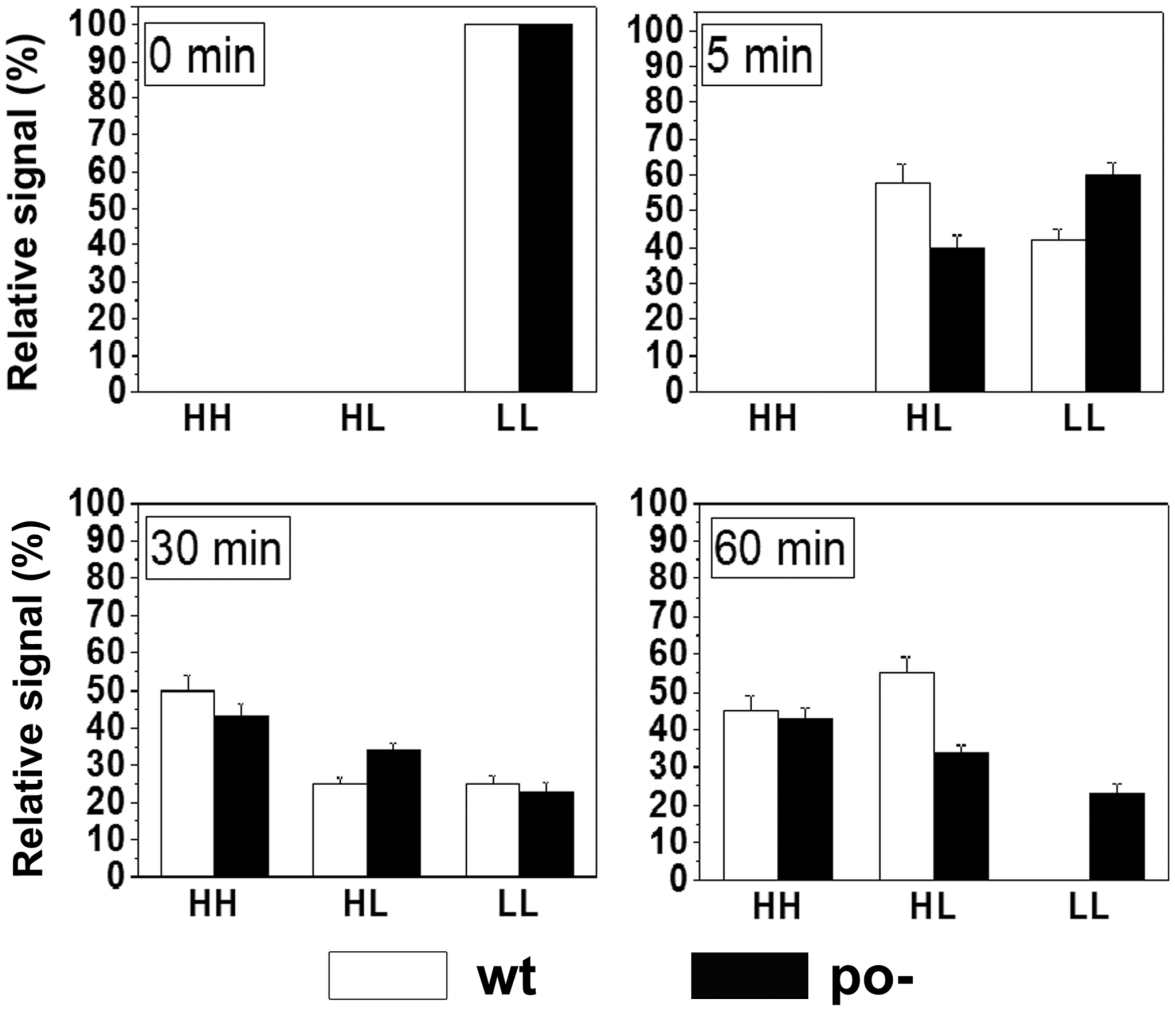
The fate of phage λ DNA in *E. coli* infected with λpapa (wt) or λ*p*_O_− (po−) as assessed by density shift experiments. After infecting bacteria with the indicated phages, further incubation was performed in the heavy medium (containing [^13^C]glucose and [^15^N]H_4_Cl) for 60 min. Samples were withdrawn at indicated times, total DNA was isolated and centrifuged in a CsCl density gradient. Signals from particular positions [fully heavy (HH), heavy-light (HL) and fully light (LL)] were estimated by hybridization of the DNA on a nitrocellulose membrane with a fluorescein-labeled probe and densitometry. Standard deviation was depicted by error bars.

Comparison of density shift experiment results, obtained after infection with wild-type λ phage or λ *p*_O_− mutant, revealed that distribution of DNA molecules between heavy-light and full-heavy position, and thus timing of the switch from θ to σ mode of replication, was similar in both cases (Figure 2). However, approximately one-fourth of the λ *p*_O_− genomes remained unreplicated, being locked in the full-light position (Figure 2). This indicates that the activity of *p*_O_ considerably affects efficiency of λ phage DNA replication, while having minor effects on the switch from θ to σ mode.

### Directionality of the λ*p*_O_− plasmid replication

**Figure 3.**
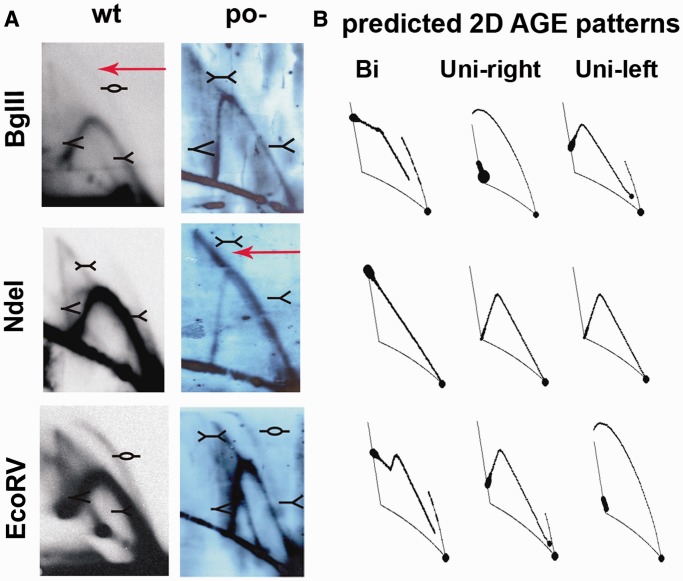
2D-AGE analysis of directionality of replication of the λ plasmid pKB2po−, in *E. coli* MG1655. Autoradiograms (**A**) and computer-simulated 2D-AGE patterns of various types of replication (**B**) are shown. Positions of DNA molecules of particular shapes are marked. Restriction enzymes used for the analysis are indicated. Arrows mark elements altered in the pKB2p_O_− plasmid in comparison with pKB2, bubble arc (BglII digest) and double Y (NdeI) signals.

Results of previous studies strongly suggested that transcription from the *p*_R_ promoter influences directionality of λ DNA replication, particularly by stimulation of bidirectional replication (22,23). Wild-type λ plasmids replicate bidirectionally and unidirectionally with similar frequencies, and within the unidirectional type, both rightward and leftward replication can be detected (47). Although we have observed the influence of the *p*_O_ promoter dysfunction on the phage DNA replication efficiency rather than the switch between the two modes, it cannot be excluded that proportions between molecules replicating in both and/or one of the directions are altered by the mutation in the *p*_O_ promoter. Therefore, we studied this possibility by using 2D-AGE of plasmid replication intermediates. As a model, we used plasmids pKB2 (wild type λ plasmid) and pKB2p_O_− (bearing the point mutation in the −10 region of *p*_O_) that harbor a region essential for λ DNA replication (Supplementary Table S2 and Figure 3).

Previously reported analysis of the pKB2 replication intermediates revealed the presence of unidirectionally and bidirectionally replicating molecules in similar amounts (47). Results of analogous experiments performed with pKB2p_O_− are presented in Figure 3 and Supplementary Figure S2. Overall amount of replicating molecules was lower relative to the wild-type pKB2 plasmid (higher amount of DNA replication intermediates had to be used to obtain the signal), corroborating the conclusion that the *p*_O_−  mutation results in a decreased efficiency of λ plasmid DNA synthesis.

Different variants of this experiment confirmed that both bidirectional and unidirectional leftward replication occur in the case of pKB2p_O_− (a map of pKB2p_O_− including restriction sites used in the analysis is shown in Supplementary Figure S1A). The latter type of replication is suggested by the presence of the bubble arc in the experiment with HindIII- and EcoRV-digested pKB2p_O_− (Figure 3). The bubble arc in the case of the EcoRV digestion confirms the existence of unidirectional leftward replication. Its absence after digestion with BglII indicates that unidirectional rightward replication is abolished in this mutant. 2D-AGE analysis after digestion with NdeI revealed the predominance of bidirectional replication over unidirectional (Figure 3).

One should note that the experimental data presented in Figure 3 differs from the theoretical pattern because the single Y arc appears on each picture of 2D-AGE, but it is not included in any of the theoretical schemes. This arc represents the replication forks migrating along DNA strands that do not contain the replication *origin* (e.g. multimeric forms of plasmids or replication intermediates sheared during the extraction procedure). The presence of this arc interferes with the comparative analysis of the theoretical schemes with images obtained as results of the experiment. In the case of the EcoRV digest, the double Y signal that is present on experimental pictures is not marked on the theoretical scheme. This signal comes from recombination intermediates, and it is not the same signal as that which appears in the scheme ‘Bi’. Generally, such signals emerging near the single Y arc signal, which comes from DNA fragments that do not contain the *origin* of replication, are more difficult to interpret owing to their overlapping. In this case, the HindIII digest is the least informative, as the *origin* of replication is situated almost in the middle of this fragment. Therefore, the choice of other restriction digests, BglII and EcoRV, in which the origin is situated at the left and at the right end of the fragment, respectively, allowed for more precise identification of the directionality of replication. Appearance or disappearance of the long bubble arc, in the case of such digests, enables detection of replication forks proceeding leftward or rightward, respectively. Consequently, the lack of this signal in the case of BglII digestion indicates the absence of plasmid molecules replicating according to the θ unidirectional rightward mode. Conversely, the bubble arc present in the case of the EcoRV digest indicates that plasmid replicates according to the unidirectional leftward mode.

To sum up, these experiments indicated that pKB2p_O_−, in contrast to the wild-type plasmid, replicates mainly bidirectionally and that unidirectional rightward replication is impaired. The observed weak replication signal of pKB2po− plasmid is also in agreement with the decreased efficiency of DNA replication of the λ phage bearing the *p*_O_− mutation, and strongly supports the hypothesis that *p*_O_ promoter’s activity plays an important role in the λ DNA replication.

### Directionality of the λ*p*_O_− plasmid replication under the conditions of enhanced transcription from the *p*_R_ promoter

**Figure 4.**
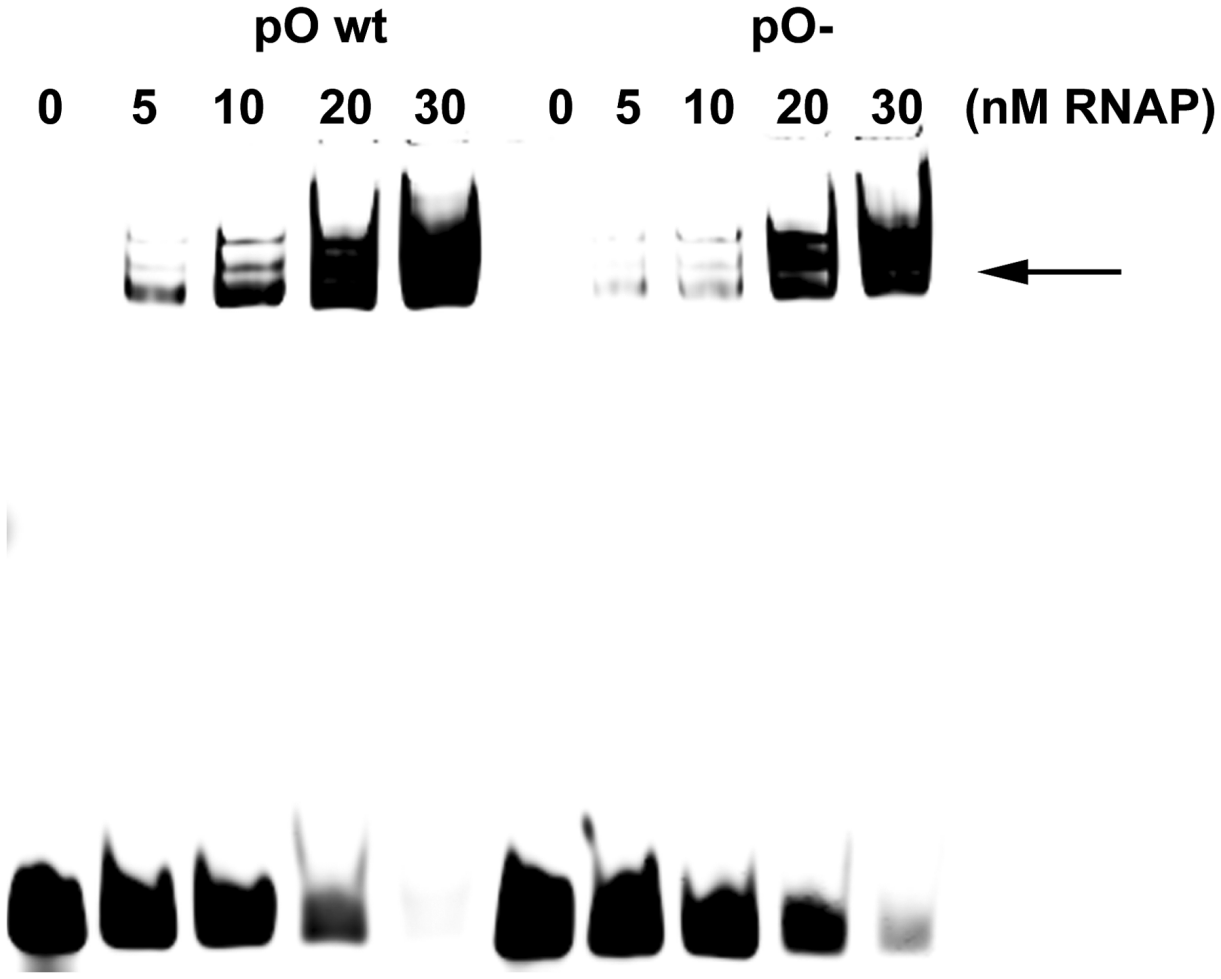
Effect of the *p*_O_− mutation on RNA polymerase biding to the *p*_O_ promoter sequence. Interaction of RNA polymerase with the promoter sequence was assessed by using electrophoretic mobility shift assay. DNA fragment, labeled with Cy5, containing the *p*_O_ sequence was incubated with indicated concentrations of RNA polymerase and resolved electrophoretically in a native polyacrylamide gel. Positions of RNA polymerase–DNA complexes are depicted by an arrow.

Analysis of the bacteriophage λ DNA replication, as well as the analysis of λ phage-derived plasmid replication described above, indicates that the *p*_O_ promoter mutation affects the efficiency and directionality of replication. The point mutation in the −10 region of the *p*_O_ promoter dramatically decreases its activity (32), and we found that this is due to reduction of efficiency of RNA polymerase binding in this region (Figure 4). As described above, replication initiating from *ori*λ is regulated by the transcription starting from the rightward *p*_R_ promoter, which affects both the efficiency and directionality of λ DNA replication (8). Therefore, we hypothesized that the observed aberrancies in the DNA replication could be a consequence of the influence of *p*_O_ activity on the transcriptional activation step starting from the *p*_R_ promoter.

**Figure 5.**
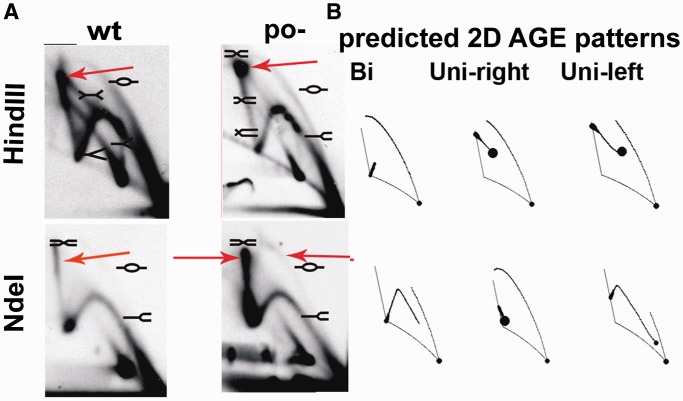
2D-AGE analysis of directionality of replication of the λ plasmids devoid of Cro repressor function, pKBlin p_O_− and pKBlin in *E. coli* MG1655. Autoradiograms (**A**) and computer-simulated 2D-AGE patterns of various types of replication (**B**) are shown. Positions of DNA molecules of particular shapes are marked. Restriction enzymes used for the analysis are indicated. Arrows mark recombination intermediates.

The Cro repressor, translated from the *p*_R_ transcript, negatively regulates transcription from the *p*_R_ promoter. It was shown previously that λ plasmid expressing a gene coding for defective Cro protein (pKBlin, Supplementary Table S2) replicates predominantly in a bidirectional manner (47). We compared directionality of replication of the pKBlin plasmid with the replication pattern of its counterpart bearing the defective *p*_O_ promoter (pKBlin p_O_−, Supplementary Table S2). 2D-AGE analysis of replication intermediates resulting from replication initiated at *ori*λ revealed that both plasmids replicate similarly, with the advantage of bidirectional replication over unidirectional replication in both directions. Interestingly, the signal of unidirectional rightward replication, visible after digestion with BglII as a bubble arc, which was absent in the case of the pKBpo− plasmid, is restored in the absence of the functional Cro protein (compare Figures 3 and 5). However, replication patterns obtained after 2D-AGE of the double *cro*^−^*p*_O_− mutants revealed also a strong signal of nonreplicating molecules with an X-shape, which are generated during DNA recombination (Figure 5). This signal is also stronger for pKBlin (*cro*^−^) plasmid in comparison with the wild-type λ plasmid (pKB2, Figure 3), but less pronounced than that observed for the double mutant (compare Figure 5, pKBlin). In addition, the general intensity of the signal of replicating molecules was comparable in the case of both versions of the pKBlin plasmid, contrary to weak signal observed for pKB2po−. Although the enhanced level of transcription from the *p*_R_ promoter suppresses the lack of the rightward unidirectional replication in the population of *p*_O_− plasmid molecules, and restores the intensity of the signal from replicating molecules in general, the mutation in the *p*_O_ promoter results in the increased population of recombination intermediates. This result may indicate that despite the increase of *p*_R_ activity alleviating a defect in the initiation of DNA replication evoked by *p*_O_ inactivity, resulting replication forks do not progress efficiently and may be repaired by recombination processes, according to previously proposed mechanism (48,49).

### The *p*_O_ promoter dysfunction affects transcription-mediated regulation of the *ori*λ activity

The results presented in the preceding paragraph imply that a functional interdependence exists between the two promoters in the regulation of replication starting from *ori*λ. Efficiency of the initiation of the λ DNA replication may, thus, depend on the balanced activity of both *p*_R_ and *p*_O_ promoters. To further investigate this possibility, we used a system in which efficiency of transcription from the *p*_R_ promoter activating *ori*λ could be controlled. Our previous results demonstrated that the *p*_R_ promoter can be replaced by an inducible promoter, and that copy number of a plasmid modified that way depends on the concentration of the inducer (50). Plasmid pTCλ5 bears the promoter of the tetracycline resistance gene (*p*_tet_), controlled by the TetR repressor, and λ replication genes *O* (together with *ori*λ located in the middle of *O*) and *P* that are positioned downstream of this promoter. Efficiency of replication of this plasmid depends on derepression of the *p*_tet_ promoter activity by tetracycline or its analogs, for instance, autoclaved chlortetracycline (aCTC) (on autoclaving, antibiotic property of chlortetracycline is lost, while its inducer feature is retained). We used this plasmid as a convenient tool to test whether eliminating transcription from *p*_O_ has an impact on transcriptional activation of *ori*λ, mediated by the *p*_tet_ activity.

**Figure 6.**
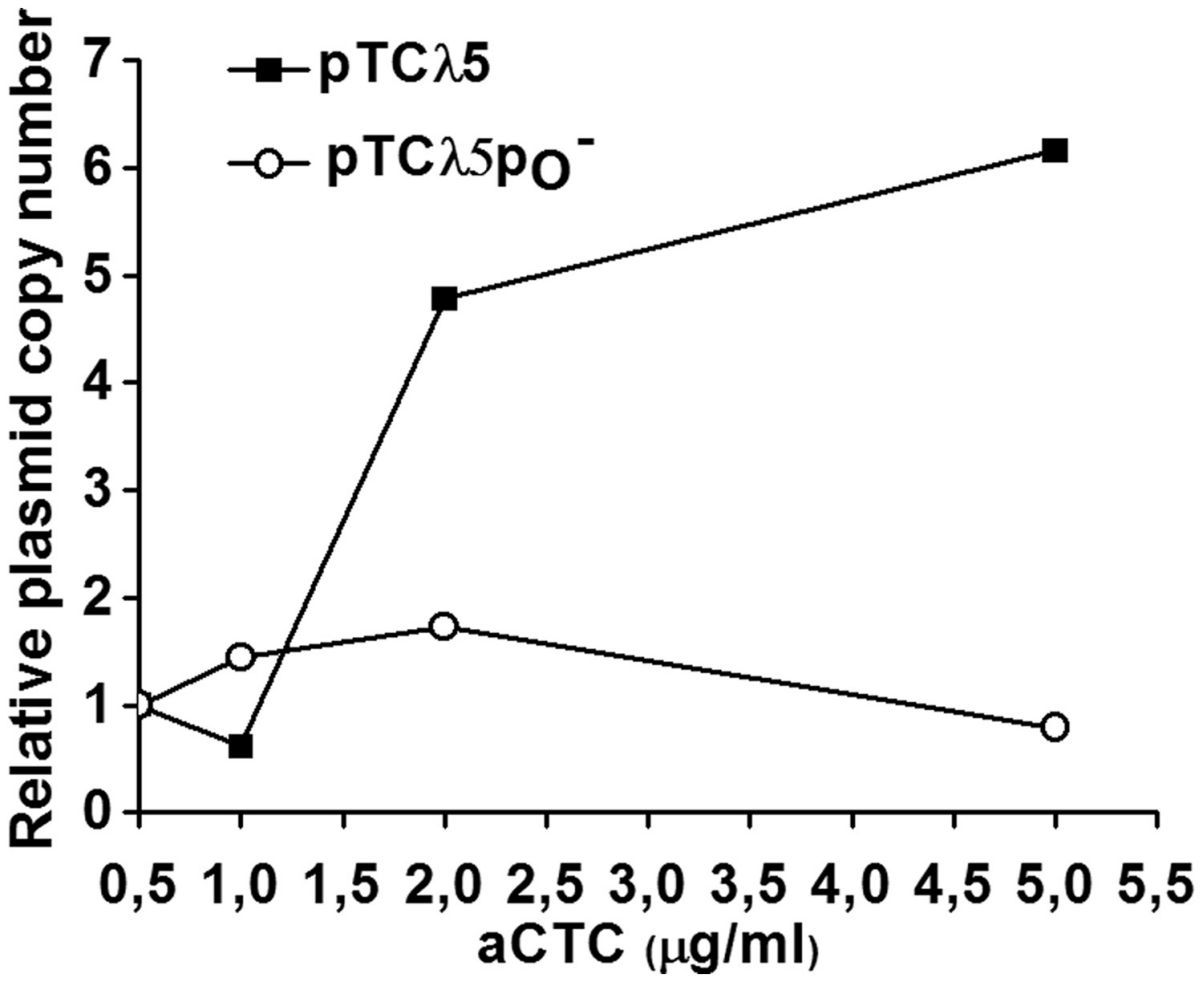
Influence of increased efficiency of transcriptional activation of *ori*λ on the copy number of plasmids pTCλ5 and pTCλ5p_O_− in *E. coli recA* cells. Bacteria were grown at 37°C in LB medium containing indicated amounts of aCTC. Copy number is presented in arbitrary counts. For each plasmid, the copy number at aCTC concentration 0.5 μg/ml was assumed as 1.

Copy number of monomeric plasmid pTCλ5 and its analog containing the *p*_O_− mutation was determined after treatment with various aCTC concentrations. The experiment was performed in *E. coli recA* cells, defective in DNA recombination to prevent formation of plasmid multimers. Importantly, characteristic regulation of the copy number in response to the inducer concentration is preserved in this strain. In the case of pTCλ5, the copy number rose with increasing aCTC concentration, as it was described previously (50). However, an increase in the amount of plasmid pTCλ5p_O_− per bacterial mass was significantly less pronounced, and at higher aCTC concentrations, even a slight decrease was noted (Figure 6).

In the *recA^+^* strain, plasmid pTCλ5 formed multimers, particularly at higher concentrations of the inducer, as it was demonstrated previously (51). Moreover, the amount of plasmid pTCλ5 monomers decreased proportionally to an increase in total plasmid copy number. In contrast, the percentage of monomeric forms of the pTCλ5p_O_− plasmid dropped considerably at higher aCTC concentrations, although the overall copy number remained low (data not shown). This observation implies that, under conditions allowing for high activity of the promoter responsible for transcriptional activation of *ori*λ, a lack of the intact *p*_O_ promoter may result in an increased plasmid multimerization. It is in agreement with results from the analysis of the pKBlin plasmid replication (Figure 5), showing an increased number of the recombination intermediates in the absence of functional *p*_O_ promoter.

Plasmid multimerization has adverse effects on its maintenance in bacterial population, resulting in the decreased plasmid stability (52). Because λ plasmids do not possess any active partitioning system, and the inheritance of plasmid copies by daughter cells is random, reduction in plasmid copy number and enhanced multimerization should be reflected by more rapid plasmid loss from cells cultured without antibiotic selection. Results of plasmid maintenance investigation demonstrated that the λ*p*_O_− plasmid, contrary to the wild-type λ plasmid, was rapidly lost from the cell culture after <20 generations of cells grown without a selective pressure (Supplementary Figure S3). This result supports earlier conclusions that decreased activity of *p*_O_ considerably impairs λ plasmid replication and the very low stability observed for the mutant plasmid may in part result from enhanced multimerization, as it was previously observed with respect to ColE1-like and other plasmids (51,52).

### Possible interference between transcription events starting from *p*_R_ and *p*_O_

**Figure 7.**
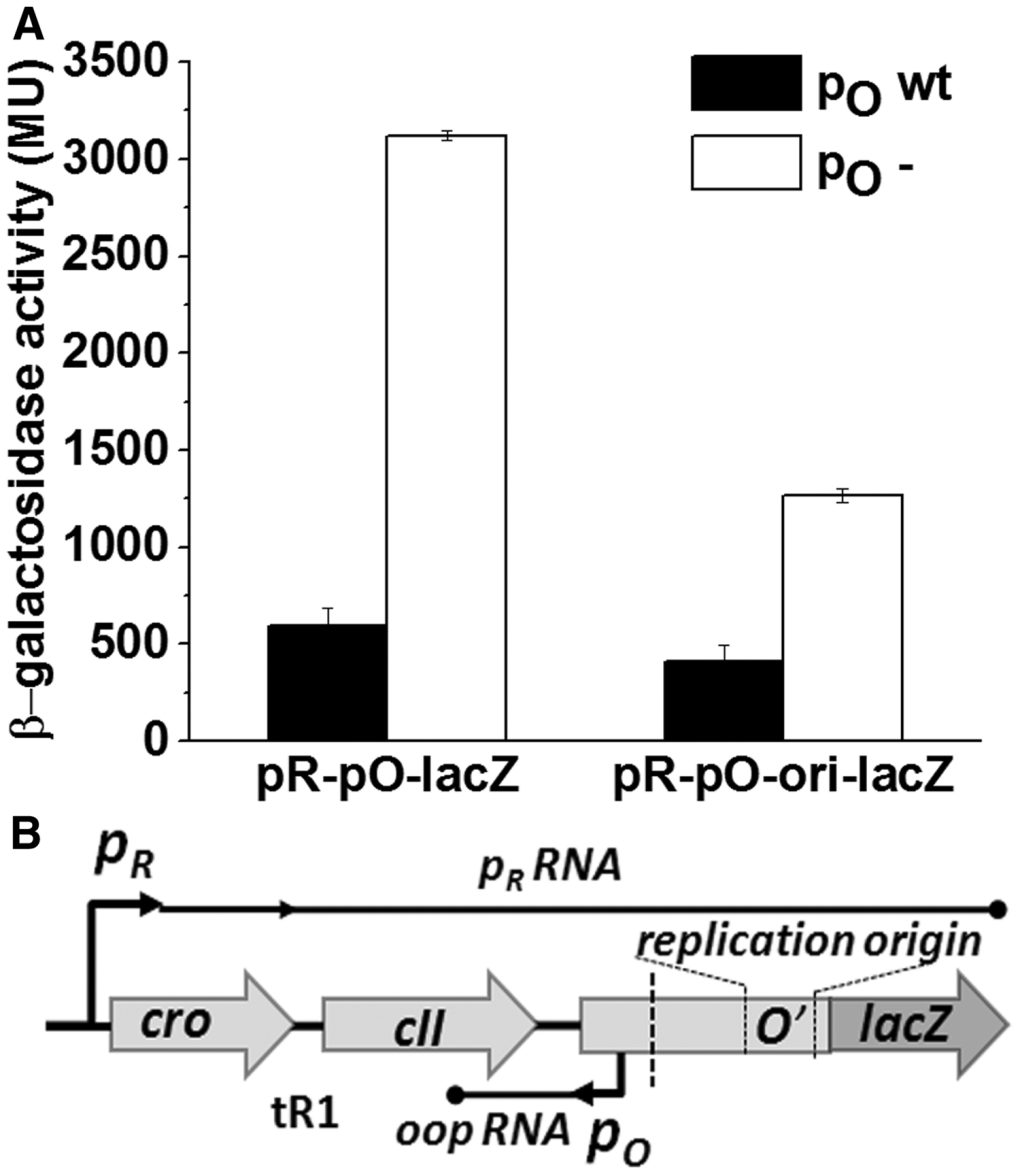
Effect of the *p*_O_ promoter activity on the efficiency of transcription starting from *p*_R_. (**A**) The level of transcription starting from *p*_R_ was assessed in MG1655Δlac strain, bearing multicopy *p*_R_-*p*_O_-*lacZ* (pTac800wt and pTac800p_O_−) and *p*_R_-*p*_O_-*oriλ-lacZ* (pTac1400wt and pTac1400p_O_−) fusions. β-galactosidase activity is presented in Miller units. (**B**) Schematic representation of the fusion constructs *p*_R_-*p*_O_-*oriλ-lacZ*. The 3′ end of the λ DNA fragment present in the *p*_R_-*p*_O_-*lacZ* construct was marked by a vertical dotted line.

Results of the experiments described so far suggest an interplay between *p*_R_ and *p*_O_ promoters’ activities, and its essential role in the replication of λ phage DNA. Therefore, we decided to investigate if the presence or absence of the *p*_O_ promoter function influences transcription starting form *p*_R_. To address this problem, we have constructed transcriptional fusions with the *lacZ* gene, containing two oppositely oriented promoters, *p*_R_ and *p*_O_, in their native distance and sequence context (Figure 7B). Organization of the used constructs results in the transcription of the *lacZ* gene originating from the *p*_R_ promoter. One of these fusions harbored, in addition, the *ori*λ region. Interestingly, in the presence of the dysfunctional *p*_O_ promoter, activity of β-galactosidase was remarkably higher than that observed for the fusion harboring the wild-type *p*_O_ sequence (Figure 7A). Similar effect was observed also in the case of the *p*_R_-*p*_O_-*ori*λ-*lacZ* fusion. These results may suggest that the decreased activity of the *p*_O_ promoter increases the amount of *p*_R_-initiated transcription elongating beyond the *p*_O_ promoter sequence. It was demonstrated previously that *oop* RNA, the transcript originating from *p*_O_, does not influence the stability of mRNA coding for the λO and λP proteins, while it acts as an antisense RNA negatively regulating the *cII* gene expression (30,31). Thus, it is unlikely that the higher efficiency of *lacZ* expression results from the stabilization of the mRNA in the absence of *oop* RNA. Moreover, although a fragment of the λO gene precedes the *lacZ* sequence in *p*_R_-*p*_O_-*ori*λ-*lacZ* constructs, *lacZ* is expressed as an independent open reading frame. Thus, its increased expression cannot be explained by the increased translation of the *O* mRNA in the absence of *oop*.

To test the influence of λO protein overproduction on the transcription starting from the *p*_R_ promoter, we used the constructs bearing *p*_R_-*p*_O_-*oriλ-lacZ* sequence configuration. We assumed that formation of the O-some structure might impede to some extent elongating transcription complex *in vivo*. While β-galactosidase activity obtained from the construct bearing the wild-type *p*_O_ promoter dropped significantly with the increased expression of *O*, the rise of the level of the initiator protein had only minor effect in the case of the version bearing the nonfunctional *p*_O_ promoter (Supplementary Figure S4). This result suggests that in the absence of *p*_O_ activity, λO might bind less efficiently to iterons present in the *ori* region. Such effect could be a consequence of enhanced transcription from *p*_R_, which might remove the O-some more effectively. Alternatively, *p*_O_ activity could directly influence the efficiency of binding or stability of the initial λO-*ori*λ complex. We conclude that, most likely either transcription from *p*_O_ or formation of the transcription complex at this promoter interferes with *p*_R_ activity, causing impairment of RNA production downstream of the latter promoter. We cannot exclude a possibility that the absence of *p*_O_ activity results in a stimulation of transcription from the *p*_R_ promoter at the initiation stage; however, this scenario seems less likely.

### The *p*_O_ dysfunction may weaken the binding of the λO protein to λ iterones

The results presented in the preceding section might suggest that decreased transcription from the *p*_O_ promoter has adverse effects on the λO binding to the iterons. Therefore, we asked if the decreased *p*_O_ promoter activity could affect the formation of λ replication complex, in consequence decreasing the efficiency of replication. In particular, we wondered whether dysfunction of the *p*_O_ promoter could influence interactions between λO and its binding sites *in vivo*. To test this, we have constructed two series of ColE1-like plasmids bearing various numbers of iteron sequences, one based on a medium copy number plasmid (pBR322) and the other on a high copy number plasmid (pUC19). We assumed that when λ plasmid DNA is introduced into cells bearing plasmids with iterons, these λO-binding sequences should outcompete iterons located on the λ plasmid in binding the replication initiator protein. This would result in impaired replication and hence reduced number of transformants obtained after transformation of *E. coli* cells by λ plasmids. Therefore, cells harboring competitor plasmids were transformed with either wild-type λ plasmid (pKB2) or its variant containing the *p*_O_− mutation (pKB2p_O_−). Assessment of the efficiency of transformation revealed that the presence of the iterons on a high copy number plasmid (the pUC19 derivative) resulted in a decreased efficiency of transformation by λ plasmid DNA in all tested experimental systems (Supplementary Table S4). Moreover, the level of transformation impairment was proportional to the number of iteron sequences present on the pUC19-derived plasmid, confirming that the effect was caused by the presence of the λO binding sites. Interestingly, effects on transformation efficiency were significantly stronger for λ plasmids devoid of the functional *p*_O_ promoter (Supplementary Table S4). Analogous experiments with pBR322-derived plasmids bearing iteron sequences revealed no significant influence on the efficiency of transformation by λ plasmids, most likely reflecting the difference in the plasmid copy number between pBR322 and pUC19. These results strongly suggest that the presence of the intact *p*_O_ promoter is important for efficient binding of the λO protein to *ori*λ present *in cis.*

**Figure 8.**
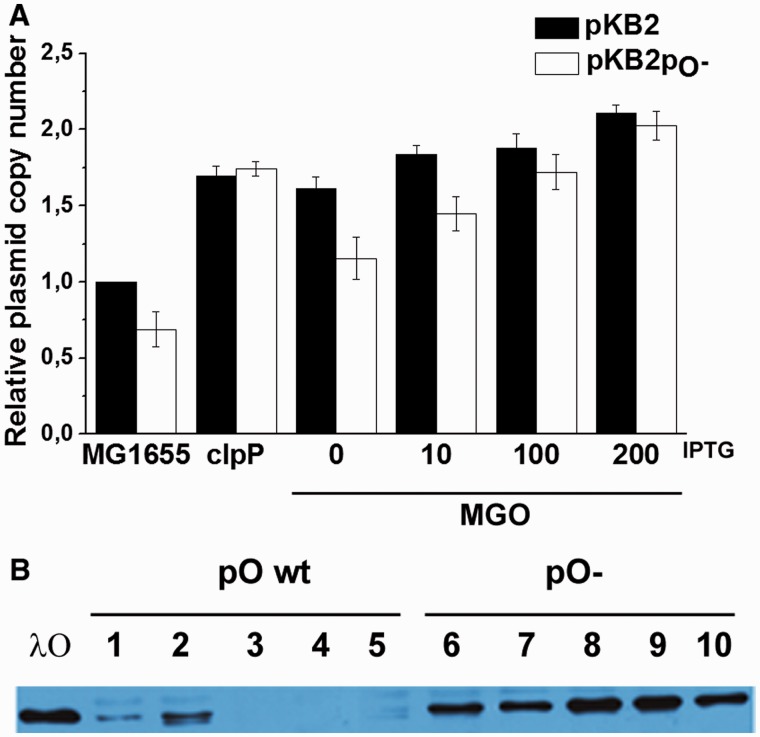
Replication of plasmid pKB2 and pKB2p_O_− in *E. coli* wt strain (MG1655), *clpP* mutant and MG1655 derivative (MGO) containing the *O* gene copy on the chromosome under the control of an IPTG-inducible promoter. (**A**) Relative plasmid amount per bacterial mass was assessed by plasmid isolation, DNA digestion with unique restriction enzyme followed by agarose electrophoresis and densitometry. IPTG concentrations used to stimulate λO overproduction are indicated (μM). (**B**) The level of λO protein present in the MG1655 *clpP* mutant, bearing λ plasmids pKB2 and pKB2p_O_−. Protein amount was assessed by immunodetection in the samples taken from overnight cultures (lanes 1 and 6) and during different phases of the culture growth (lanes 2–5 and 7–10).

If this hypothesis is true, one should expect that a decreased copy number of λ*p*_O_− plasmid could be corrected in cells containing increased amounts of the λO protein. We addressed this question by measuring relative levels of DNA of λ plasmids, either wild-type (pKB2) or with the *p*_O_− mutation (pKB2p_O_−), in the wild-type host bearing the *O* gene under the control of an IPTG-inducible promoter on the chromosome (strain MGO, Supplementary Table S1). We found that pKB2p_O_− copy number was significantly decreased relative to that of the wild-type λ plasmid in the absence of the *O* gene expression *in trans*. However, on *O* expression induction, this parameter was significantly increased for both plasmids (Figure 8A). Furthermore, the effect was proportional to the level of λO production. Efficiency of overproduction of λO under these conditions was estimated by western blotting with antibodies specific for this protein (data not shown).

To further examine this phenomenon, we aimed to compare the relative levels of both plasmids in the wild-type and *clpP* hosts. The latter strain is devoid of ClpP, a component of the protease, which specifically degrades λO (53). Thus, the level of the λ phage replication initiator protein is significantly increased in this bacterial strain (54). We found that the copy number of both plasmids was considerably higher in the *clpP* mutant in comparison with the wild-type strain, abolishing the copy number difference between the pKB2 and pKB2p_O_− plasmids (Figure 8A). However, a closer examination of the level of the λO protein expressed from each of the plasmids in the *clpP* strain revealed that cells harboring the mutant plasmid contain significantly higher amounts of λO (Figure 8B). Therefore, the impairment of λ plasmid replication in the absence of the intact *p*_O_ promoter cannot be explained by a decreased efficiency of the *O* gene expression. Importantly, λO protein is synthesized from the *p*_R_-derived transcript; thus, we conclude that levels of this RNA are increased in cells harboring pKB2p_O_− plasmid. This result is also in agreement with observed increased expression of *lacZ* from the *p*_R_-*p*_O_::*lacZ* fusion in the absence of *p*_O_ function. Nevertheless, these results together with competition experiments indicate that the *p*_O_− mutation affects formation of the replication complex at *ori*λ. This defect can be alleviated by increased amount of λO protein delivered *in trans* (Figure 8A, MGO) or when the protein is stabilized in the *clpP* strain (Figure 8A clpP). In addition, results of these experiments suggest that formation of the initiation complex is the rate-limiting step in λ DNA replication.

### Effect of the *p*_O_ promoter positioning relative to *ori*λ on the efficiency of λ plasmid replication *in vivo* and *in vitro*

One of the questions emerging from the results of experiments presented so far is whether *p*_O_ activity exerts an effect on the λO-*ori*λ complex formation directly or through its effect on transcription starting from *p*_R_. We addressed this problem by investigating the impact of positioning of the *p*_O_ promoter with respect to *ori*λ on the λ DNA replication. Therefore, we constructed a series of plasmids containing insertions, which increased the distance between *p*_O_ and *ori*λ by 6, 10, 50, 100, or 500 bp. Because the *p*_O_ promoter is located at the 5′ end of the *O* gene, all DNA manipulations in this region disrupted the coding sequence for the λ replication initiator. Therefore, we introduced the desired insertions into plasmids containing both *ori*λ and ColE1-type *origin*. In addition, a part of the gene coding for the C-terminal fragment of the λO protein was removed from this plasmid, to eliminate the possibility of replication initiation by the λO protein produced *in cis*.

We compared the efficiency of transformation of *polA*^+^ and *polA* strains with the modified plasmids (in the latter strain, the replication starting from ColE1-like *ori* is abolished). In both hosts, the λO protein was provided *in trans* from a helper plasmid (pLamberA). Results of the experiments presented in Table 1 demonstrated that even a small increase (6 or 10 bp) in the distance between the *p*_O_ promoter and *ori*λ resulted in a drastic reduction in the efficiency of *polA* mutant transformation by the plasmids carrying such modifications. This suggests that not only the process of transcription *per se* but also RNA polymerase binding at the specific position relative to *ori*λ is important for efficient replication of λ plasmids. Alternatively, the formation of a specific DNA structure or nucleoprotein complex is required for the replication. One may expect that such minor changes in the promoter-*origin* relative location should not be so significant if *p*_O_-mediated regulation of *ori*λ replication relied only on possible effect of the ongoing transcription on the DNA topology at the *origin* (for instance, by introducing a defined number of negative supercoils). Therefore, these results imply that the role of *p*_O_ in the formation of the λ replication complex may not be restricted to the regulation of *p*_R_-derived transcription.

**Table 1.** Efficiency of transformation of *E. coli* polA^+^ and polA1 strains by double-origin (λ-ColE1) plasmids bearing various insertions between pO and oriλ

Plasmid	Efficiency of transformation (transformants per 1 µg of DNA)^a^	
	*polA*^+^	*polA1*
pdelλO	4.5 × 10^4^	2.9 × 10^3^
pdelλOpo-	2.7 × 10^4^	<1 × 10^0^
pdelλOins6	2.8 × 10^4^	2.3 × 10^1^
pdelλOins10	4.0 × 10^4^	1.7 × 10^1^
pdelλOins50	3.6 × 10^4^	<1 × 10^0^
pdelλOins100	3.6 × 10^4^	<1 × 10^0^
pdelλOins500	2.9 × 10^4^	<1 × 10^0^

The results represent the mean value of three independent experiments.

^a^C600 and C600polA strains were used as recipients. Each host bore a helper plasmid pLamberA.

Results of *in vitro* replication assays performed using the series of mutated plasmids as templates confirmed that the *p*_O_ promoter mutation or increasing its relative distance to the *origin* have a negative effect on λ plasmid replication. The *p*_O_ dysfunction resulted in ∼40% decrease in the efficiency of replication (Supplementary Figure S5). Replication of plasmids containing larger insertions (100 and 500 bp) was also less effective than that observed for the wild-type plasmid, although the effects of those modifications were less pronounced than those observed in the *in vivo* studies (Supplementary Figure S5).

## DISCUSSION

It has been shown in several studies that RNA polymerase activity plays an important role in the regulation of bacteriophage λ DNA replication at the initiation stage (8). The *p*_R_ promoter was identified as a source of transcriptional activation of *ori*λ, but the exact molecular mechanism of this phenomenon has not been resolved (8). However, studies demonstrating that λO replication initiator enhances transcription-induced supercoiling by DNA gyrase and has an ability to form topologically isolated domain suggested a mechanism based on the changes in DNA topology introduced by RNA polymerase (21,55). It was also proposed that the efficiency of λ DNA replication depends on the activity of another promoter, *p*_O_, present in the vicinity of the *origin* and directed oppositely to *p*_R_ (27, 56–58). *p*_O_ drives synthesis of a short antisense transcript, which regulates stability of the cognate *cII* mRNA via RNase III-dependent mechanism. Despite these early proposals (27, 56–58), *oop* RNA seems dispensable for the replication *in vitro*, and its increased stability does not exert an effect on the λ plasmid DNA synthesis *in vivo* (31,44). Thus, the function of the *p*_O_ promoter in the λ DNA replication remained obscure.

In this study, by using mutants containing inactive *p*_O_ promoter, we confirmed the influence of its activity on the efficiency of λ plasmid replication. Importantly, we have also shown that the presence of the dysfunctional *p*_O_ impedes the initiation of λ phage DNA replication, proving that this promoter plays a role during the lytic cycle, in the natural genetic context of the virus (Figure 2). In attempt to identify the mechanism of *p*_O_ action, we demonstrated that it affects also directionality of this process. Interestingly, the lack of *p*_O_ activity resulted in the advantage of the bidirectional replication, and this effect was similar to that observed previously for plasmids with increased activity of *p*_R_ (45). Taking into account this result and the respective positioning of *p*_R_ an *p*_O_, which may result in their interference (59), we hypothesized that *p*_O_ activity may influence transcription started at the *p*_R_ promoter. By employing transcriptional fusions of *p*_R_ with the *lacZ* reporter gene, containing either wild-type or defective *p*_O_ promoter sequence, we have shown that the presence of the functional *p*_O_ promoter affects *p*_R_-driven gene expression (Figure 7). This result was corroborated by the increased amount of λO (produced from the *p*_R_ transcript) observed in the *clpP* strain transformed with pKB2p_O_− plasmid, in comparison with the one bearing wild-type pKB2 (Figure 8B). These data suggest that the role of *p*_O_ may rely on tuning of the level of transcription from *p*_R_, which reaches the *origin* of replication. We propose that this control mechanism operates via a direct interference of the RNA polymerases transcribing in the opposite directions. This kind of regulation has been demonstrated for a number of other convergent promoters (59), and it could result from RNA polymerase pausing or dissociation on the collision. Alternative explanation might involve the role of antisense *oop* RNA in the posttranscriptional regulation of the *p*_R_-driven mRNA level. However, it has been shown that *oop* does not alter the stability of λ*O-P* mRNA; hence, it has no effect on the fate of the part of transcript downstream of *p*_O_ (31). In agreement with the proposed role of the *p*_O_ promoter, our results demonstrated that the impact of the increased level of transcription from *p*_R_ on λ plasmid replication was dependent on the activity of *p*_O_. Namely, in the recombination-deficient *recA* strain, the increase in the activity of the *p*_tet_ promoter, substituting for *p*_R_, led to a substantial drop in the pTCλ5p_O_− plasmid copy number, contrary to its counterpart containing wild-type *p*_O_ (Figure 6). In addition, in the absence of *p*_O_, elevated level of transcription from the *p*_R_ promoter resulted in enhancement of recombination processes (Figure 5) and plasmid multimerization (these results will be discussed in the next section).

What would be the consequences of the increased level of the *p*_R_-initiated transcription passing through the *ori*λ for the initiation of the λ DNA replication in the light of the proposed mechanism? Such more frequent transcription events would result in a higher level of the λO and λP replication proteins and, possibly, enhanced activation of the λ replication initiation complex. Hence, this could potentially lead to overinitiation at the *ori*λ and problems with the replication fork progression, due to their collision, as it was proposed, for instance, for over-initiating DnaA mutants of *E. coli* (60). Interestingly, in our studies we observed increased multimerization and enhanced level of recombination processes taking place in the case of plasmids bearing the inactive *p*_O_ promoter (Figure 5 and Supplementary Figure S2). These effects, resulting most probably from the SOS response induction, could support the above-mentioned hypothesis, as replication forks collision is accompanied by frequent double-strand breaks and activation of the repair mechanisms (60). Nevertheless, other results presented in this work suggest that the initiation of λ DNA replication is less efficient in the presence of the defective *p*_O_ promoter (Figure 2), and that the excess of the λO protein can suppress the negative effect of the lack of *p*_O_ activity on λ plasmid copy number (Figure 8). Moreover, plasmids bearing *p*_O_− mutation are more sensitive to the presence of additional λO-binding sequences provided *in trans* than their wild-type counterparts (Supplementary Table S4). To sum up, these results disfavor over-initiation and suggest that, on the contrary, the formation of the λ replication initiation complex might be hampered in the absence of *p*_O_ activity. In addition, it was demonstrated previously that the presence of the defective *p*_O_ exerts adverse effect also on the replication of λ plasmid DNA, which was initiated by a replication complex inherited by one of the daughter copies (58). This mode of replication can be observed in the absence of protein synthesis, when λO, which is unprotected by the components of the replication complex, is rapidly degraded (8). Thus, the excess of λO produced from a *p*_O_− plasmid cannot be a sole explanation of its replication deficiency.

Inefficient assembly of the initiation complex, resulting from the *p*_O_ promoter defect, can also be explained by the excessive transcription from *p*_R_, reaching the *origin* of replication. Namely, it was demonstrated that the λO-*ori*λ complex, which forms at the first stage of the replication initiation, is destabilized by the action of RNA polymerase, and liberated λO is hydrolyzed by the ClpXP protease (26). Subsequent assembly of the pre-primosomal complex, consisting of λO-λP-DnaB, protects λO from the RNA polymerase-dependent proteolysis (26). Thus, many transcription events reaching the λ *origin* may, in the presence of ClpXP protease, result in an inefficient formation of the O-some structure. Similarly, transcription directed into *oriC* from the *mioC* promoter was shown to negatively regulate initiation of *E. coli* chromosomal DNA replication (61). In addition, such transcription events were demonstrated to interfere with replication starting from autonomously replicating sequence in *Saccharomyces cerevisiae* (62), and hinder binding of a regulatory protein (63). The proposed negative influence of transcription from *p*_R_ on the λ replication complex formation seems to be in opposition to the restoration of the unidirectional rightward replication by the inactivation of the Cro repressor, demonstrated in the case of pKBlin po- plasmid (Figure 5). The lack of Cro function results in around 2-fold increase in the efficiency of transcription starting from the *p*_R_ promoter (64), but the impact of upregulation of *p*_R_ activity on the replication complex assembly may be counterbalanced by the enhanced production of the λO protein. Importantly, replication of plasmids devoid of Cro function was shown to be cell cycle-dependent, contrary to their wild-type counterparts (65). Thus, other mechanisms may also be responsible for the suppression of the effect of the *p*_O_− mutation on the unidirectional rightward replication in the absence of Cro activity.

Enhanced frequency of transcription passing through the replication region could also account for the increase in the abundance of recombination intermediates and multimerization observed for the plasmids bearing the *p*_O_ mutation (Figure 5 and Supplementary Figure S2). It was shown previously that a high level of activity of the rightward promoter caused drastic stimulation of multimer formation by λ plasmid, and its instability in bacterial population, even in the presence of the intact *p*_O_ promoter (50,51). Elevated recombination activity, resulting in multimerization, indicates induction of the SOS response, and is a hallmark of replication forks aberrations (66). Thus, it is possible that inactivation of *p*_O_ not only hinders assembly of the replication initiation proteins at *ori*λ, but also perturbs progression of the formed replication complexes. This could result from interference of transcription and replication machineries. Such encounters were demonstrated to take place *in vivo* and cause activation of DNA repair processes and genomic instability (49,67). Both head-on and co-directional collisions were shown to exert deleterious effects on replication forks (49,67). Increased amount of trailing RNA polymerases, which initiated at *p*_R_, may affect λ replication complex directly or by introducing unfavorable changes in DNA topology.

One of the most enigmatic finding of our studies was that changing the distance between the *p*_O_ promoter and *ori*λ exerts a strong negative effect on the λ plasmid replication *in vitro* and *in vivo* (Table 1). These changes decreased the efficiency of the plasmid DNA synthesis *in vitro* to a similar degree as did *p*_O_− mutation (Supplementary Figure S4), indicating that positioning of the promoter is important for its function in the replication control. One plausible explanation would involve interaction between the *p*_O_ promoter and *ori*λ, most likely mediated by proteins bound to these sequences. Recently, we have shown that λO interacts directly with RNA polymerase (24) what would make both these proteins possible candidates to mediate such a complex formation. Although by increasing the distance between *p*_O_ and *ori*λ we also introduced additional gap between *p_R_* and the replication initiation site, the latter change was shown to have little impact on the λ DNA replication (68).

In this study, we did not investigate how the activity of *p*_O_ promoter is regulated; however, in the light of the proposed mechanism, periodic changes in accordance with the initiation of replication may be assumed. Indeed, *p*_O_ was previously shown to be upregulated during the period of the virus DNA replication in a manner dependent on the components of λ replication complex (69).

A separate problem is a possibility that the *p*_O_-initiated transcript, *oop* RNA, might influence replication initiation from *ori*λ. Dependence of the inhibition of λ phage DNA replication by plasmids bearing a fragment of λ DNA and preexisting in the infected cell on the plasmid-borne *oop* sequence was recently reported (70). When in our experiments *oop* was overexpressed from a plasmid, it strongly decreased efficiency of replication of both wild-type λ plasmid and its *p*_O_− counterpart (our unpublished results). However, in the same set of experiments, the presence of the empty vector (used otherwise to *oop* expression) altered the ratio between the wild-type and mutated λ plasmids. On the other hand, no influence of the considerably increased *oop* RNA levels (obtained due to *pcnB* mutation and increased stability of this transcript) on the copy number of both wild-type and *p*_O_− λ plasmids was observed (44). These results suggest that *oop* RNA *per se* has little importance in the natural regulation of replication from *ori*λ, and that the inhibition of λ DNA replication by *oop* overexpressed from a plasmid might be specific to such experimental system.

In summary, we propose that in the compact genome of bacteriophage λ, a dual promoter system, consisting of *p*_R_ and *p*_O_, has evolved to ensure coordination of transcription and replication processes.

## SUPPLEMENTARY DATA

Supplementary Data are available at NAR Online.

## FUNDING

Ministry of Science and Higher Education (Poland) [N301 4140 33 to M.G.] and the National Science Center (Poland) [2011/02/A/NZ1/00009 to G.W.]. Funding for open access charge: National Science Center (Poland) [2011/02/A/NZ1/00009 to G.W.].

*Conflict of interest statement*. None declared.

## Supplementary Material

Supplementary Data
