# Supplementary material for: A dual promoter system regulating λ DNA replication initiation
Source: Nucleic Acids Res. 2014 Feb 5;42(7):4450–62. doi: 10.1093/nar/gku103 (PMC3985674; doi:10.1093/nar/gku103)
Supplement: Supplementary Data [file supp_42_7_4450__index.html]

A dual promoter system regulating λ DNA replication initiation — A dual promoter system regulating λ DNA replication initiation — Supplementary Data 

# A dual promoter system regulating λ DNA replication initiation

## Supplementary Data

files

**Files in this Data Supplement:**

- Supplementary Data - pdf file
