# Supplementary material for: A dual promoter system regulating λ DNA replication initiation
Source: Nucleic Acids Res. 2014 Feb 5;42(7):4450–62. doi: 10.1093/nar/gku103 (PMC3985674; doi:10.1093/nar/gku103)
Supplement: Supplementary Data [file supp_gku103_nar-02913-v-2013-File010.pdf]

Supplemental material

Content:

I. Supplemental Materials and methods
Table 1-3
Strains and plasmids construction
II. Supplemental results
Figures S1-S5
Table S4

I. Supplemental Materials and Methods

Table 1. *Escherichia coli* strains

Strain	Relevant genotype	Reference
C600	*surE44 hsdR thi-1 thr-1 leuB6 lacY1 tonA21*	(70)
C600polA1	As C600 but *fadA::* Tn10 *polA1*; constructed by P1 transduction from H221 *zfi3134::Tn10kan*	this work
CAG18562		(71)
DH5α	*supE44* Δ*lacU169* [□80 *lacZΔM15*] *hsdR17 recA1 gyrA96 thi-1 relA1*	(72)
H221	*fadA::*Tn*10 polA1*	(73)
MM294	*supE44 hsdR endA1 pro thi*	(74)
MG1655	wild type	(75)
MGO	as MG1655 but containing pCAHlamO plasmid integrated in the lambda attachment site	this work

Table 2. Plasmids

Plasmid	Characteristics and/or construction	Reference
pKB2	Wild-type λ plasmid bearing a *kan* gene	(76)
pKB2po-	As pKB2 but bearing a point mutation in the -10 region of the p_O promoter	(58)
pLamber	A hybrid plasmid bearing *oriλ* and *ori*ColE1-like, and *kan* and *bla* genes	(73)
pLamberA	As pLamber but lacking the *kan* gene; constructed by removing of the 916 bp *Hin*dIII-*Nco*I fragment from pLamber	This work
pLamberB	As pLamber but lacking the *bla* gene; constructed by removing of the 803 bp *Asu*II-*Ase*I fragment from pLamber	This work
pdelλO	A derivative of pLamberB bearing a deletion of a part of the *O* gene, coding for C-terminal domain of λO.	This work
pdelλOpo-	As pdelλO but bearing a point mutation in the -10 region of the p_O promoter	This work
pdelλOins6, pdelλOins10, pdelλOins50, pdelλOins100, pdelλOins500	A series of plasmids derived from pdelλO, in which insertions of 6, 10, 50, 100 or 500 bp were introduced between p_O and *ori*[illegible]	This work
pIK12	pBluescript II SK vector bearing [illegible] O^+ and *P*ts1[illegible]A66 alleles	(77)
pEW1	As pIK12 but bearing wild-type alleles of *O* and *P*; constructed by replacement of the *Eco*RI- *Sst*II fragment from pIK12 with an	(44)

	analogous fragment of pKB2	
pGP1-2	A part of the T7 RNA polymerase expression system	(78)
pUC19oriλ	pUC19 bearing oriλ	this work
pUC19it34	pUC19 containing λ iterons 3 and 4	this work
pUC19it4	pUC19 containing λ iteron 4	this work
pBRoriλ	pBR322 bearing oriλ	this work
pBRit34	pBR322 containing λ iterons 3 and 4	this work
pBRit4	pBR322 containing λ iteron 4	this work
pCAH56	contains *attP* of the λ phage, ptac promoter and *cat* gene	(79)
pCAHlamO	pCAH56 derivative bearing the O gene under the control of ptac promoter	this work
$pINT^{ts}$	a helper plasmid overexpressing λ phage integrase, used for integration of pCAH56 plasmid derivatives with the host chromosome	(79)
pKBlin	as pKB2 but devoid of Cro function	(45)
pKBlinp_O-	as pKBlin but p_O-	This work
pTCλ5	p_{tet} , *tetR*, λ replication region under control of p_{tet}	This work
pTCλ5p_O-	as pTCλ5, but bearing p_O- mutation	This work
pTCλ1	p_{tet} , *tetR*, λ replication region under control of p_{tet}	(49)
pUC19		(80)
pBR322		(81)
pTAC3734	*lacZ, ampR*	(82)

Table 3. Oligonucleotides

No.	Primer sequence
1.	5' CGACCTGACAAACACAGACTGGAT
2.	5' CACTTGCTGCCGCTCTGAATTGC
3.	5' GATGGTTACGCCGAGCTCAGACTATCAAATATG
4.	5' CATATTTGATAGTCTGAGCTCGGCGTAACCATC
5.	5' GAGGCTTATTCGTCTAGAGCGCGGCGCAGATCTG
6.	5' CAGATCTGCGCCGCGCTCTAGACGAATAAGCCTC.
7.	5' AGAGGTAACTTTGCCCTCGAGGAGCGTAATGTGGCA
8.	5' TGCCACATTACGCTCCTCGAGGGCAAAGTTACCTCT
9.	5' CTTGCTCGAGTAGTCGTCGTATCTG
10.	5' GGTCTAGATTCAGCCACTGCAT TTCC
11.	5' GATTCTCGAGTTCTCAATGCTG
12.	5' CCTGTGCACGATTTAGAGGTCT
13.	5'- CACCGATTCTCAACTTAGCGAGATTAC
14.	5'-CTGGCTGATGGTGCGATAGTCTTC
15.	5'-CGGGATCCTCTAGACATCCCTCAAATTGGGGGA TTGCTATCCCTCAAAACAGGGGGACGGATCCGC
16.	5'-CGGGATCCTCTAGAATCCCTCAAAACAGGGGGAGGATCCGC
17.	5'-AGGAGTCCATATGACAAATACAGCAAAAATACTCAACTTCGGCAGAGG
18.	5'-GCGGGATCCTCATAGATCCACCCCGTAAATCCAG
19.	5'-GGGAGAAATATCTGGATCCGTGCGTGTTGAC
20.	5'-CCGAGCGTTCTGAACAAAGCTTATGGAGTTCTG
21.	5'-CTGGTCAGAAGCTTCGCCAGAATTCTCTGACG

Strains and plasmids construction

E. coli strains are listed in Table S1 and plasmids are described in Table S2. Bacteriophages λpapa (from our collection) and λp_O- (30) were used in density shift experiments. Phage P1*vir* (from our collection) was used for transduction.

MGO strain was constructed by integration of the plasmid pCAHlamO (Table S2) into the lambda attachment site using lambda site-specific recombination (79).

Plasmids pLamberA and pLamberB were constructed by deletion of fragments, containing *kan* or *bla* gene, respectively, from pLamber plasmid (73), as described in the Table 2. Both those plasmids contain λ phage region encompassing p_R promoter and genes *cro*, *cII*, *O* and *P*, as well as ColE1-type origin of replication.

Plasmid pdelλO was constructed by PCR mutagenesis. Primers 1 and 2 (Table S3) were used in the reaction leading to deletion of the 291 bp fragment from pLamberB, coding for the C-terminal domain of the λO protein.

Plasmid pdelλOpo- was constructed by ligation of the 5.8 kb *Hin*dIII- *Eco*RI fragment of pdelλO and the 1.584 bp *Hin*dIII-*Eco*RI fragment of pKB2po- .

A series of pdelλO-derived plasmids bearing insertions of 6, 10, 50, 100 or 500 bp, called pdelλOins6, pdelλOins10, pdelλOins50, pdelλOins100 and pdelλOins500, respectively, were constructed as follows. 6 bp and 10 bp insertions were introduced into pdelλO plasmid by PCR using following pairs of primers: 3 and 4 or 5 and 6 (Table S3). Plasmids bearing 50 bp and 100 bp insertions were constructed in two steps. First, a unique *Xho*I site was introduced into a pdelλO plasmid by PCR mutagenesis, using primers 7 and 8 (Table S3). Then, hybridizations of two pairs of the 50 bp-long and 100 bp-long oligonucleotides, whose sequences corresponded to fragments of the spectinomycin-resistance gene, were performed to obtain dsDNAs. Following phosphorylation of the 5'-ends by T4 polynucleotide kinase (1 unit, Promega), the double stranded oligonucleotides were ligated with pdelλO, previously

linearized with *Xho*I. The 500 bp fragment for construction of pdelλOins500 was PCR-amplified using a template containing spectinomycin-resistance gene and primers 9 an 10 (Table S3). Following digestion with *Xho*I and *Xba*I, the 500 bp fragment was ligated with a *Xho*I-linearized pdelλOins10 plasmid. The series of plasmids based on pdelλO does not express a functional replication intiator O protein.

Plasmids pUC19 (80) bearing various numbers of iterons were constructed by cloning the below listed DNA fragments into BamHI site of pUC19: pUC19oriλ contains a PCR product obtained with primers 13 and 14 (Table s3), pUC19it34 contains a fragment obtained by hybridization of the oligo 15 (Table S3) and its complementary counterpart and subsequent BamHI digest, pUC19it4 bears a fragment obtained by hybridization and BamHI digest of the oligo 16 (Table S3) and its complementary counterpart. Derivatives of pBR322 with λ iteron sequences (pBRoriλ, pBRit34, pBRit4) were constructed analogously.

Plasmid pCAHlamO was constructed by cloning a PCR fragment containing the O gene sequence, obtained using primers 17 and 18 (Table S3), into BamHI-NdeI sites of plasmid pCAH56 (79).

Plasmids containing p_R-p_O::*lacZ* (pTac800) and p_R-p_O-*oriλ*::*lacZ* (pTac1400) fusions were constructed by inserting a PCR fragment, amplified using primers 19 and 20 (pTac800wt and p_O-) or 19 and 21 (pTac1400 wt and p_O-), into BamHI-HindIII sites of pTac3734 (82) (Table S2).

pTCλ5 and pTCλ5p_O^- plasmid was cloned by inserting 1727 bp fragment (obtained by PCR, using primers 11 and 12 (Table S3) and plasmid pKB2 or pKB2p_O^-, respectively, as a template), into XhoI-ApaL1 sites of plasmid pTCλ1 (49).

β-galactosidase assay

Overnight cultures were diluted in the LB medium with or without indicated concentrations of the inducer and grown until OD578=0.2. After reaching desired density, cultures were chilled on ice. 100µl of culture were added to 1.5ml of the Z buffer and 100 µl of chloroform and vortexed. Subsequently, solution was incubated for 10 minutes. Following incubation, 400 µl of the ONPG solution was added and the reaction was carried out for 5 minutes. The reaction was stopped by addition of 1ml of 1M sodium carbonate. The absorbance of the solution was measured at 420nm wavelength. β-galactosidase activity was calculated as described (82).

Preparation of Fraction II

E. coli C600 strain was grown in LB medium at 37˚ to OD_{600} = 1.0. The cells were collected by centrifugation, washed once in buffer A (25 mM Hepes- KOH pH 7.6, 5 mM EDTA) and then resuspended in buffer A (2 ml buffer A per 1 ml of cell paste). The cell paste was transferred to a polypropylene tube, quickly frozen in liquid nitrogen and stored at –70˚. After thawing at 0-4˚, the liquid was adjusted to 150 mM KCl and 2mM DTT, and lysozyme was added to 0.5 mg/ml. Following incubation at 0˚ for 30 min, the cells were frozen again in liquid nitrogen, thawed at 0-4˚ and centrifuged in Beckman 50.2 Ti rotor at 30,000 rpm for 30 min at 4˚. The supernatant was collected and ammonium sulfate (0.28 g per 1 ml of the cleared lysate) was slowly added at 0˚ with stirring over a 60 min period. The precipitate was collected by centrifugation at 23,000 rpm for 20 min at 4˚, pellet was resuspended in 100 µl of buffer B (25 mM Hepes – KOH pH 8.0, 0.1 mM EDTA, 2 mM DTT) and dialyzed against 200 ml of buffer B at 0˚ for 30 min. The fraction was frozen in aliquots in liquid nitrogen and stored at –70˚.

***In vitro* DNA replication**

The standard reaction mixture (final volume equal to 25 µl) consisted of: Hepes- KOH pH 7.6, 40 mM; magnesium acetate, 11 mM; bovine serum albumin, 50 µg/ml; creatine phosphate, 40 mM;

creatine kinase, 20 μg/ml; ATP, 2mM; GTP, CTP, UTP each 500 μM; dATP, dGTP, dCTP, and dTTP, each 100 μM, with [^{3}H]dTTP (150 cpm/pmol); PEG 6000, 8.6 μg/ml; supercoiled DNA, 250 ng; λO protein, 300 ng; λP protein, 80 ng. The mixtures were assembled at 0°. The reactions were initiated by addition of 1.5 μl of Fraction II, and incubation was carried at 32˚ for 2 h. Total nucleotide incorporation was measured by determining radioactivity in a scintillation counter after trichloroacetic acid precipitation.

Density shift experiments

Amount of bacteriophage particles added to bacterial cultures was monitored by titration of the λwt and λp_O- phage lysates on the strain used in this experiment. Bacteria were grown in a "light" minimal medium (84) overnight at 37˚, and - after dilution (1:50 v/v) with fresh medium - the cultivation was prolonged to OD_{500} eqal to 0.2. The bacteria were collected by centrifugation, washed with TM buffer (10 mM Tris-HCl pH = 7.2, 10 mM $MgSO_4$) and resuspended in 0.1 volume of this buffer. After 60 min incubation at 37˚, a phage lysate was added to m.o.i. = 10 and incubation was continued for 15 min. The suspension was sedimented, resuspended in the same volume of prewarmed (to 37˚) "heavy" minimal medium (containing [15N]H_4Cl and [13C]glucose instead of NH_4Cl and glucose, respectively), and incubation was carried on at 37˚. Samples of the infected culture were withdrawn at indicated times, total DNA was isolated and ultracentrifuged in the CsCl density gradient as described previously (Węgrzyn et al., 1995). Fractions were collected from the bottom of the tube, DNA was transferred to a nitrocellulose membrane and detected by hybridization with a fluorescein-labeled probe consisting of a replication region of phage λ genome. The probe was obtained by random priming reaction using Gene Images Random Prime Labeling Kit (Amersham Biosciences) and a linearized λ plasmid (pKB2). Signals were detected using Gene Image CDP-Star Detection Kit-to anti-fluorescien-AP conjugate (Amersham).

Hybridization signals were quantified densitometrically, and percent of □ DNA at each position (full-light, heavy-light, and full-heavy) was calculated.

II. Supplemental results

A

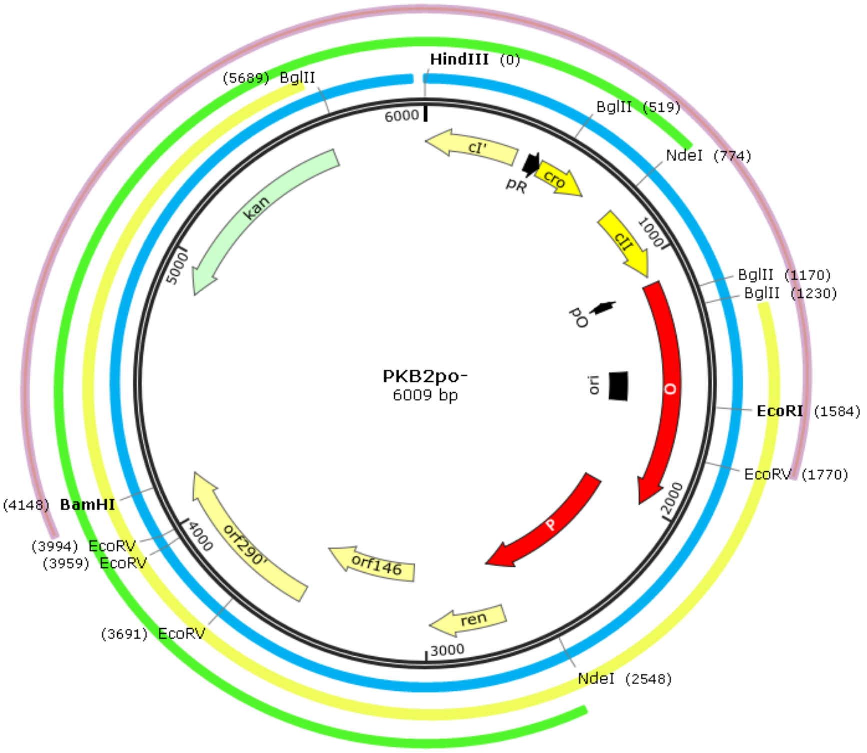

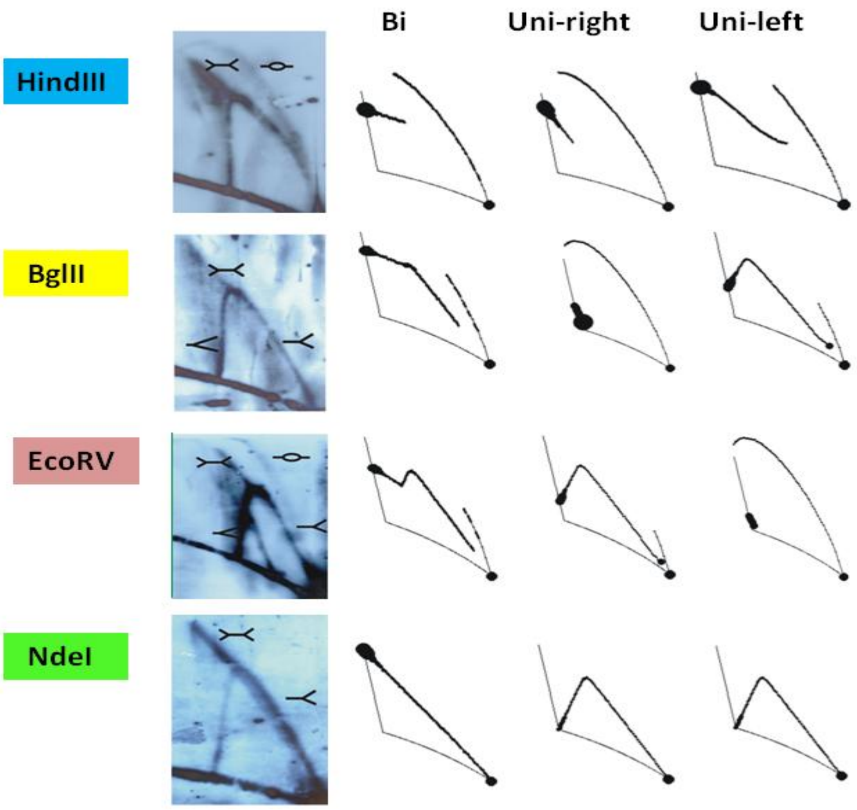

Fig. S1. A map of the pKB2p$_{O}$- plasmid. Restriction sites used in the experiments are marked and DNA fragments used as probes are shown as external circles (A), theoretical schemes and results of two-dimensional agarose gel electrophoresis of pKB2p$_{O}$- (B).

A

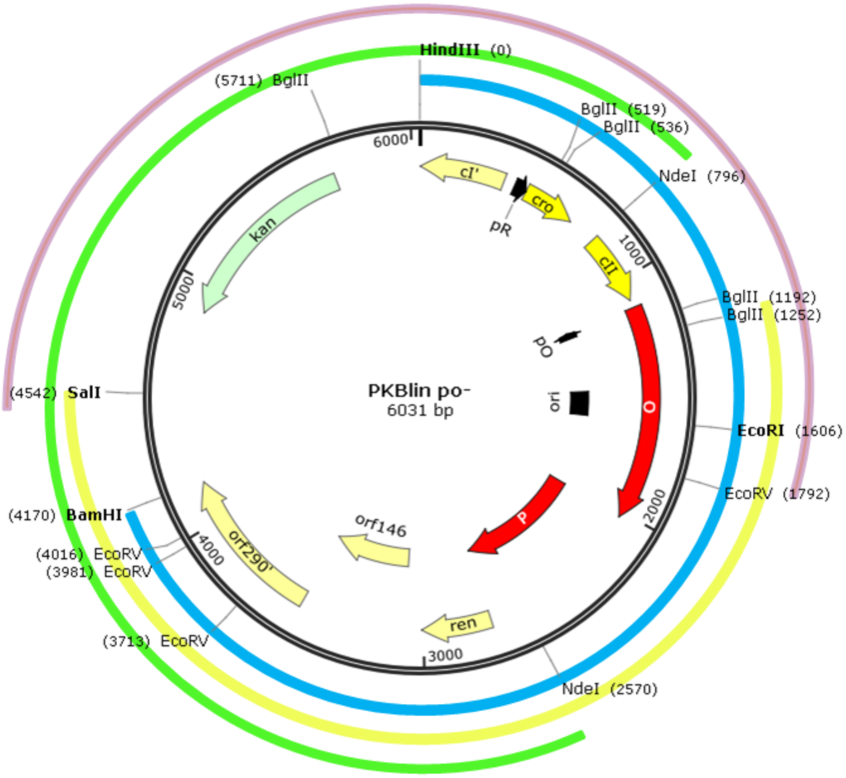

HindIII (0)
(5711) BglII
BglII (519)
BglII (536)
NdeI (796)
BglII (1192)
BglII (1252)
EcoRI (1606)
EcoRV (1792)
NdeI (2570)
(3713) EcoRV
(3981) EcoRV
(4016) EcoRV
(4170) BamHI
(4542) SalI
PKBlin po-
6031 bp
6000
1000
2000
3000
4000
5000
cI'
pR
cro
cII
pO
ori
O
P
ren
orf146
orf290'
kan

B

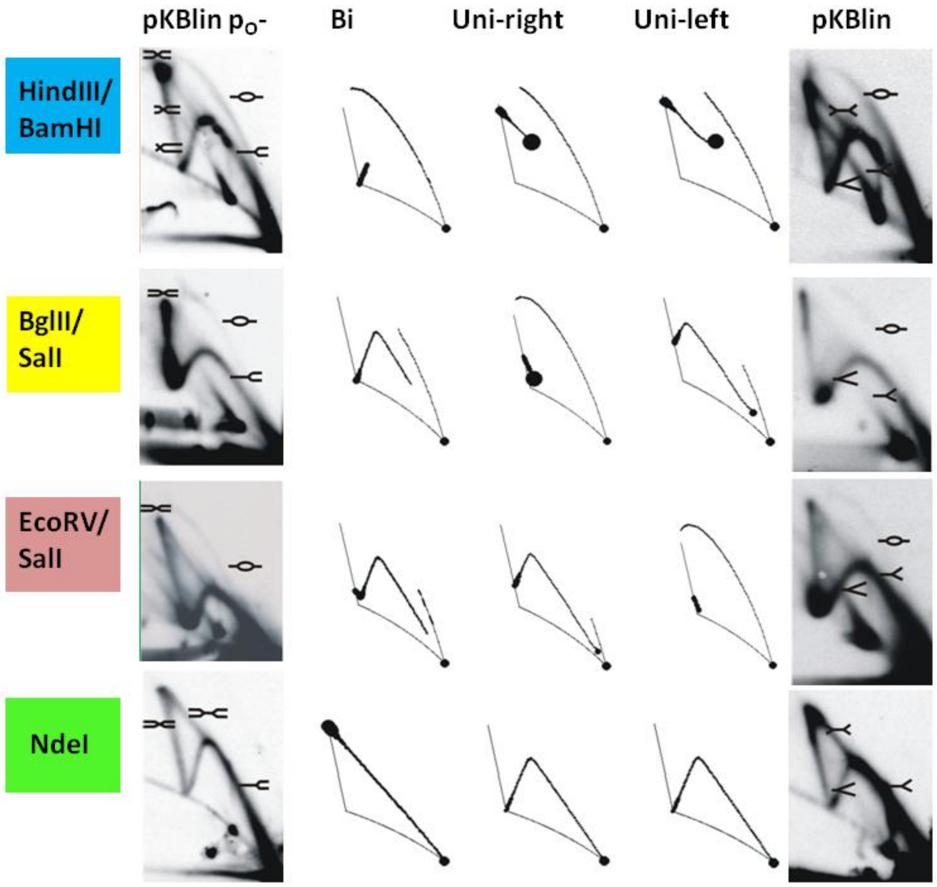

Fig. S2. A map of the pKBlin p_O- (the same as for pKBlin) plasmid. Restriction sites used in the experiments are marked and DNA fragments used as probes are shown as external circles (A), theoretical schemes and results of two-dimensional agarose gel electrophoresis of pKBlin p_O- in comparison to pKBlin (B).

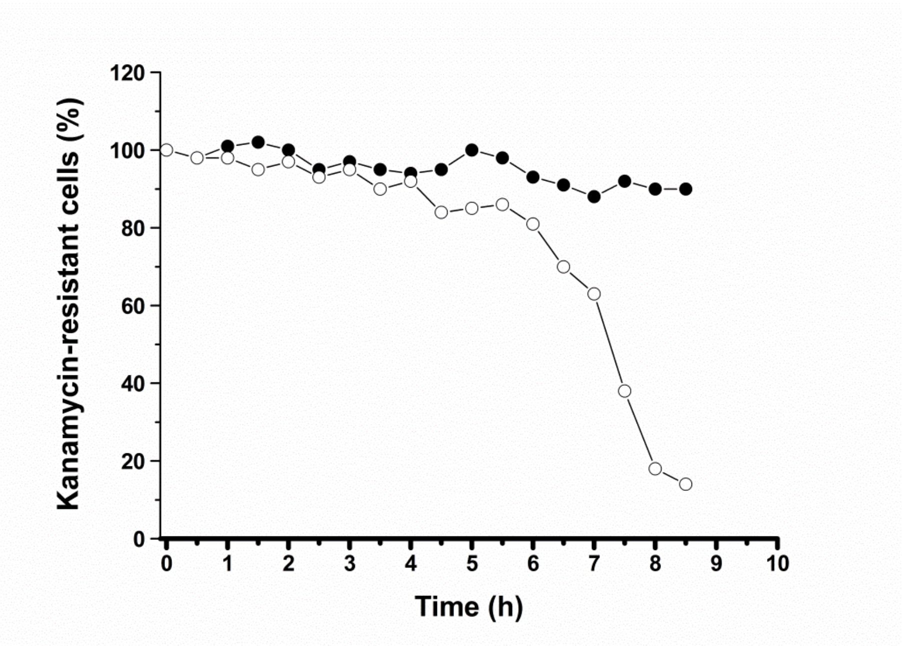

Fig. S3. Maintenance of pKB2 (wild-type λ plasmid; closed circles) and its p_O- derivative (pKB2 po-; open circles) in *E. coli* MG1655 strain. Plasmid-bearing bacteria were grown in LB medium at 37˚ without an antibiotic (overnight cultures were supplemented with 25 µg/ml kanamycin as the *kan* gene is present on both plasmids). During growth, the culture was successively diluted in a fresh LB medium and maintained in early-middle log phase. Samples were withdrawn at indicated times and percent of kanamycin-resistant cells was calculated by titrating bacteria on LB plates with and without kanamycin.

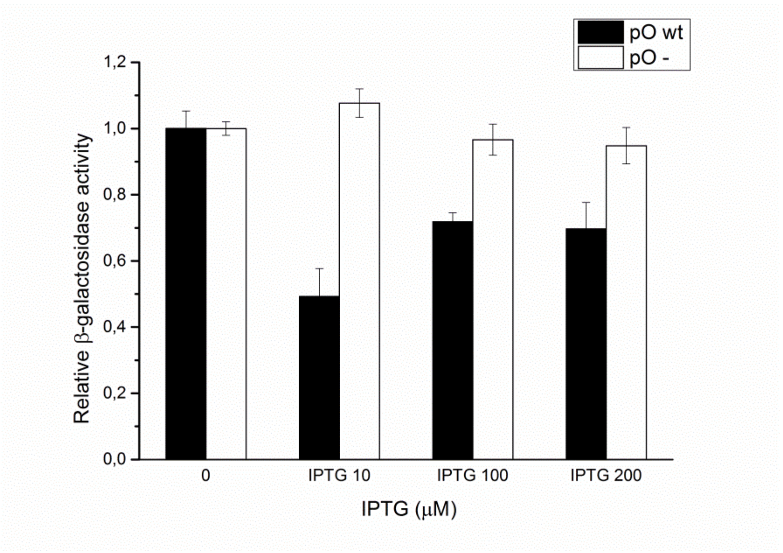

Fig. S4. Effect of pO promoter activity on the efficiency of transcription starting from p_R in the presence of oriλ sequence and the λO protein. p_R promoter activity was assessed in the presence and absence of p_O function using a strain overexpressing the λO protein (MGO), bearing p_R-p_O-*oriλ-lacZ* (pTac1400wt and pTac1400p_O-) fusions. Activity of the promoter was presented relatively to the value obtained in the absence of IPTG, which induced overproduction of λO. β-galactosidase activity is presented in Miller units

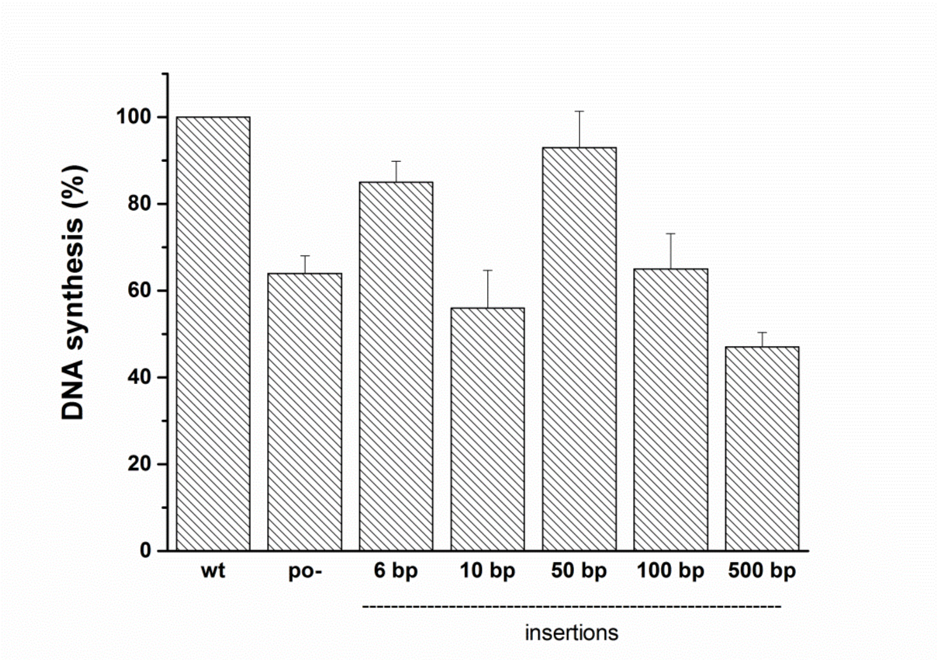

Fig. S5. *In vitro* replication of pdelλO plasmid (marked as wt) and its derivatives, bearing either the p_O- mutation (pdelλOpo-, marked as po-) or insertion of 6 (pdelλOins6, marked as 6

bp), 10 (pdelλOins10, marked as 10 bp), 50 (pdelλOins50, marked as 50 bp), 100 (pdelλOins100, marked as 100 bp) and 500 (pdelλOins500, marked as 500 bp) base pairs. The reaction was performed using Fraction II, as described in Materials and Methods. Synthesis of each hybrid λ -ColE1 replicon was estimated in the presence or absence of λO and λP proteins in the replication mixture. Thus, level of λ plasmid DNA replication was calculated as a difference between levels of DNA synthesis in these two experimental systems. The value of 100 % corresponds to 73 pmoles of synthesized DNA. Standard error was depicted by error bars.

Table S4. Efficiency of transformation of *E. coli* MG1655 strains with wild-type (pKB2) and p_O^- (pKB2p_O^-) version of λ plasmid. MG1655 strain bore pUC19 (pUC19oriλ, pUC19it34, pUC19it4) or pBR322 (pBRoriλ, pBRit34, pBRit4) derivatives with four, two or one iteron sequence of the λ origin. The results represent the mean value of three independent experiments.

MG1655/plasmid	Efficiency of transformation (transformants per 1 μg of DNA)		Ratio pKB2p_O^-/pKB2 (%)
	pKB2	pKB2p_O-	
-	5.4 (±0.01) x 10^4	3.5 (±0.10) x 10^4	66
pUC19	4.3 (± 0.13) x 10^4	2.7 (±0.14) x 10^4	61
pUC19 1 iteron	1.7 (±0.11) x 10^4	7.9 (±0.42) x 10^3	46
pUC19 2 iterons	7.8 (± 2.0) x 10^3	2.6 (±0.48) x 10^3	33
pUC19 4 iterons	1.8 (± 0.14) x 10^3	3.2 (±0.84) x 10^2	17
pBR322	1.6 (±0.02) x 10^5	9.6 (±0.83) x 10^4	62
pBR322 1iterons	8.8 (± 0.09)x 10^4	6.1 (±0.17) x 10^4	69
pBR322 2 iterons	8.3(±0,77) x 10^4	4.8 (±0.45) x 10^4	58
pBR322 4 iterons	9.7(±0.08) x 10^4	5.4 (±0.29) x 10^4	55

Supplementary references:

71. Appleyard,R.K. (1954) Segregation of new lysogenic types during growth of a doubly lysogenic strain derived from *Escherichia coli* K12. *Genetics.*, **39,** 440-452.

72. Singer,M., Baker,T.A., Schnitzler,G., Deischel,S.M., Goel,M., Dove,W., Jaacks,K.J., Grossman,A.D., Erickson,J.W., Gross,C.A. (1989) A collection of strains containing genetically linked alternating antibiotic resistance elements for genetic mapping of *Escherichia coli*. *Microbiol Rev.*, **53**, 1-24.

73. Hanahan,D. (1983) Studies on transformation of *Escherichia coli* with plasmids. *J Mol Biol.*, **166**, 557-80

74. Glinkowska,M., Majka,J., Messer,W., Węgrzyn,G. (2003) The mechanism of regulation of bacteriophage λ p_R promoter activity by *Escherichia coli* DnaA protein. *J Biol Chem.*, **278**, 22250-22256.

75. Meselson,M., Yuan,R. (1968) DNA restriction enzyme from *E. coli. Nature.*, **217**, 1110-4.

76. Jensen,K.F. (1993) The *Escherichia coli* K-12 "wild types" W3110 and MG1655 have an rph frameshift mutation that leads to pyrimidine starvation due to low *pyrE* expression levels. J Bacteriol., **175**, 3401-7.

77. Kur,J., Górska,I., Taylor,K. (1987) *Escherichia coli dnaA* initiation function is required for replication of plasmids derived from coliphage lambda. *J Mol Biol.*, **198**, 203-10.

78. Konieczny,I., Marszałek,J. (1995) The requirement for molecular chaperones in lambda DNA replication is reduced by the mutation pi in lambda P gene, which weakens the interaction between lambda P protein and DnaB helicase. *J Biol Chem.*, **270**, 9792-9.

79. Tabor,S. Richardson,C.C. (1985) A bacteriophage T7 RNA polymerase/promoter system for controlled exclusive expression of specific genes. *Proc Natl Acad Sci USA.*, **82**, 1074-8.

80. Haldimann,A., Wanner,B.L. (2001) Conditional-replication, integration, excision, and retrieval plasmid-host systems for gene structure-function studies of bacteria. *J Bacteriol.*, **183**, 6384-93.

81. Yanisch-Perron,C., Vieira,J., Messing,J. (1985) Improved M13 phage cloning vectors and host strains: nucleotide sequences of the M13mp18 and pUC19 vectors. *Gene.*, **33**, 103-19.

82. Bolivar,F., Rodriguez,R.L., Greene,P.J., Betlach,M.C., Heyneker,H.L., Boyer,H.W., Crosa, J.H., Falkow,S. (1977) Construction and characterization of new cloning vehicles. II. A multipurpose cloning system. *Gene.*, **2**, 95-113.

83. Miller,J.H. (1972) Experiments in Molecular Genetics. Cold Spring Harbor New York: Cold Spring Harbor Laboratory Press.

84. Schneider,K., Beck,C.F. (1986) Promoter-probe vectors for the analysis of divergently arranged promoters. *Gene.*, **42**, 37-48.

85. Wegrzyn,G., Taylor,K. (1992) Inheritance of the replication complex by one of two daughter copies during λ plasmid replication in Escherichia coli. *J Mol Biol.*, **226**, 681-688.
